# Swift and portable detection of *soybean mosaic virus* SC7 through RNA extraction and loop-mediated isothermal amplification using lateral flow device

**DOI:** 10.3389/fmicb.2024.1478218

**Published:** 2025-01-03

**Authors:** Shui-Xian Guo, Qing Zhang, Nan-Nan Bai, Pei-Yao Yue, Jing-Ping Niu, Cong-Cong Yin, Ai-Qin Yue, Wei-Jun Du, Jin-Zhong Zhao

**Affiliations:** ^1^Department of Basic Sciences, Shanxi Agricultural University, Taigu, Shanxi, China; ^2^College of Agronomy, Shanxi Agricultural University, Taigu, Shanxi, China; ^3^College of Life Science, Shanxi Agricultural University, Taigu, Shanxi, China

**Keywords:** soybean, *soybean mosaic virus*, virulent strain SC7, magnetic beads-based RNA extraction method, LAMP, lateral flow device

## Abstract

The soybean mosaic disease—caused by the *soybean mosaic virus* (SMV)—significantly impacts soybean quality and yield. Among its various strains, SMV-SC7 is prevalent in China. Therefore, rapid and accurate diagnosis is deemed critical to mitigate the spread of SMV-SC7. In this study, a simple and rapid magnetic bead-based RNA extraction method was optimized. Furthermore, a reverse-transcription loop-mediated isothermal amplification (RT-LAMP) assay that requires no specialized equipment such as PCR Amplifier was proposed, employing a lateral flow device (LFD) for visual interpretation of SMV-SC7. The RT-LAMP-LFD approach facilitated specificity testing of SMV-SC7. Moreover, the limit of detection (LOD) of this method was as low as 10^−5^ ng (2.4 copies). The sensitivity of RT-LAMP-LFD was 10-fold higher than that of the colorimetric RT-LAMP method. In 194 field samples tested, the RT-LAMP-LFD detection of the SMV-SC7 had accuracy of 98.45% in comparison to RT-qPCR. In conclusion, the assay exhibited high specificity, sensitivity, and rapidity, enabling economical and portable detection of SMV-SC7 and providing technical support to identify SMV-SC7-infected soybeans.

## 1 Introduction

Globally, soybean mosaic disease—caused by *Soybean mosaic virus* (SMV)—leads to decline in soybean seed yield and quality (Liu et al., 2016; Usovsky et al., 2022). Currently, effective measures to prevent SMV-associated diseases are lacking (Hajimorad et al., 2018; Usovsky et al., 2022; Song et al., 2024). Therefore, early and accurate detection of SMV is beneficial to effectively control soybean mosaic disease.

Plant virus detection primarily encompasses phenotype screening, protein analysis, and nucleic acid detection (Debreczeni et al., 2011; Fox and Mumford, 2017; Sheveleva et al., 2018). Among these methods, nucleic acid detection is particularly crucial for plant virus identification and diagnosis, owing to its exceptional sensitivity (Rubio et al., 2020). Nucleic acid amplification-based detection of plant diseases comprises three primary stages: extraction/purification of nucleic acids, amplification, and detection of the amplified product (Paul et al., 2020). The nucleic acid extraction plays a critical role in the detection process. To date, extraction from plant samples has primarily been performed using chemical reagents and silica columns (Palani et al., 2019; Paul et al., 2020). Traditional chemical-based methods are relatively cumbersome, time-consuming, and impractical for rapid on-site processing (Simões et al., 2013). Although silica gel column-based commercial kits have significantly streamlined the extraction process and yielded purer products, the higher cost of the specific kits and the consumables required makes them potentially difficult to implement in situations with limited resources or space (Smuts et al., 2014; Valiant et al., 2024). Recently, a magnetic bead (MB)-based nucleic acid extraction method has been developed and applied across various fields, offering benefits, such as simplicity, speediness (reduced extraction time by 66.7%), cost-effectiveness (saved costs by ~78.6%) (Fei et al., 2022; He et al., 2021; Li et al., 2017; Mao et al., 2024).

Nucleic acid testing plays a vital role in effectively controlling the spread of viruses. RT-qPCR was considered the gold standard to diagnose a wide range of diseases in laboratories and clinics (Yi et al., 2024). However, this method relied on expensive and sophisticated equipment. In recent years, nucleic acid-based isothermal amplification methods, such as recombinase polymerase amplification (RPA) (Ghosh et al., 2018, 2020), helicase-dependent amplification (HDA) (Vincent et al., 2004), rolling-circle amplification (RCA) (Lizardi et al., 1998), and loop-mediated isothermal amplification (LAMP) (Notomi et al., 2000; Kokane et al., 2020), have been widely used. While the RPA reaction can be performed at 42°C, it requires the addition of magnesium acetate prior to amplification, which complicates the test protocol (Behrmann et al., 2020). The HDA is successfully performed at 37°C, but it is limited by low speed and processivity of the *Escherichia coli* UvrD helicase, and lacks coordination between the helicase and the DNA polymerase (Lee et al., 2022; Wharam et al., 2001). Nevertheless, the RT-LAMP assay is straightforward. The typical requirement for such an assay is a solution mainly comprising the Bst DNA polymerase with strong strand displacement activity and tolerance for elevated temperatures, primer sets and reaction buffer. In addition, LAMP is realized by 4–6 specific primers to target 6–8 regions on a target sequence, which can ensure high specificity for target amplification (Notomi et al., 2000). Whereas both RPA and HAD are realized by two specific primers, and it is difficult to ensure high specificity. Therefore, LAMP has been widely applied across various fields due to its high simplicity and specificity including diagnosing clinical conditions, conducting veterinary tests, assessing food safety, and evaluating the environment (Dong et al., 2022; Lee et al., 2019; Sheu et al., 2018; Singh et al., 2019).

To visualize LAMP results, methods, such as turbidity measurement, DNA intercalating dyes, pH indicators, and metal ion indicators, have been employed (Bhat et al., 2013; Rahman et al., 2022; Wang et al., 2021; Wu et al., 2022; Zeng et al., 2021). However, these techniques are non-specific and may lead to false positives (Luo et al., 2021). For instance, the amplification process produces hydrogen ions along with the release of pyrophosphate (PPi), allowing visualization of the LAMP reaction by monitoring pH changes (Zhang et al., 2014). In recent years, lateral flow nucleic acid biosensors have gained significant attention due to their high sensitivity and rapid visual detection capabilities (Ge et al., 2014). LAMP results can be visualized by employing a lateral flow device (LFD) when the 5' ends of internal primers (FIP and BIP) and loop primers (LF and LB) are labeled with biotin and FAM, respectively (Peng et al., 2021). Moreover, some studies have combined LAMP with lateral flow biosensors using gold nanoparticles for the sequence-specific detection of pathogenic microorganisms (Chen et al., 2023; Wang et al., 2022). Furthermore, previous research results suggested that the integration of RT-LAMP with LFD can enhance the sensitivity of traditional RT-LAMP assays (Andrade and Lightner, 2009; Nimitphak et al., 2008; Thongkao et al., 2015).

**Figure 1:**
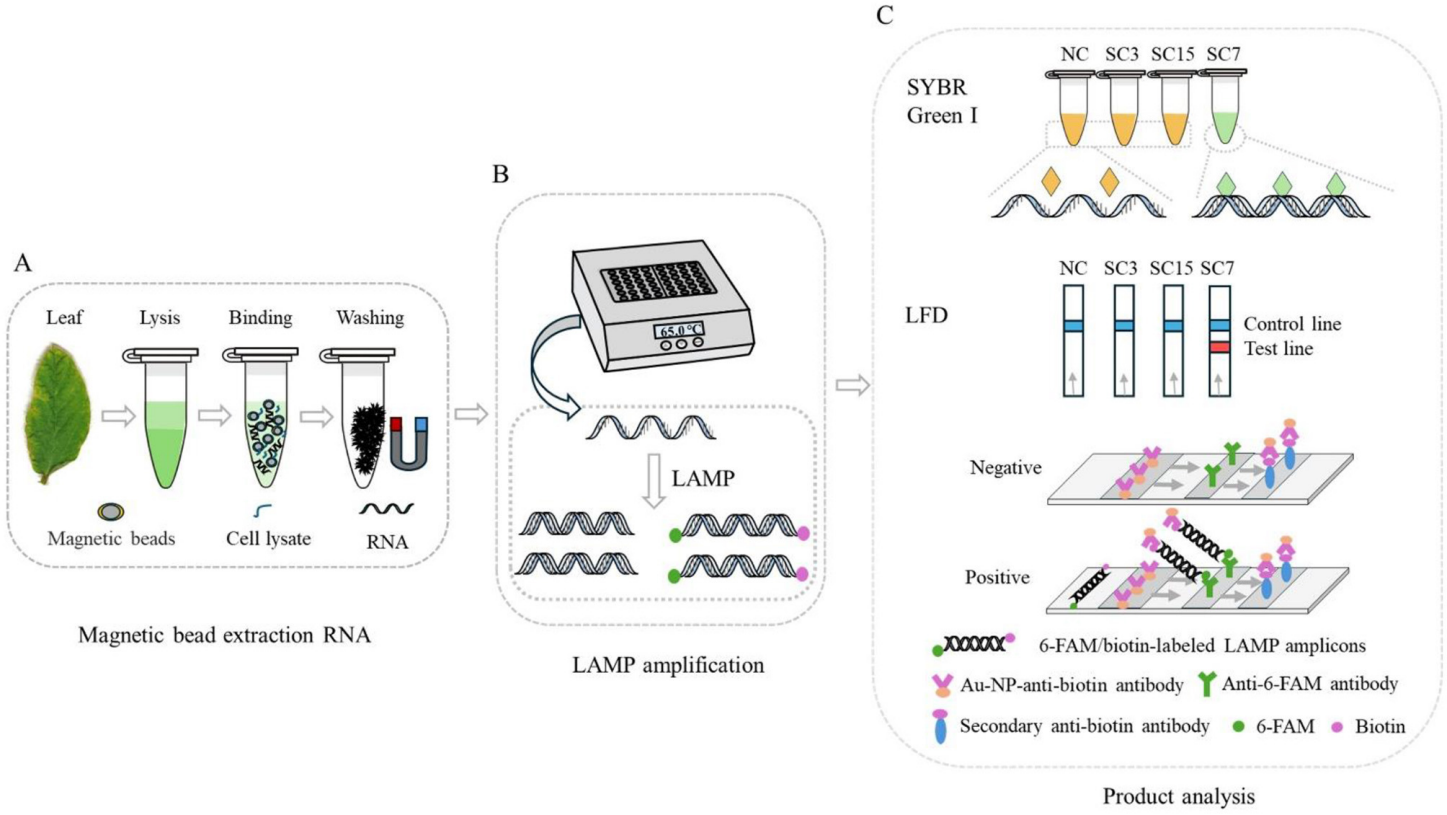
Schematic illustration of RT-LAMP-LFD principle for the detection of SMV-SC7 in soybean leaves. **(A)** Simple and fast RNA extraction with Si-OH MBs. **(B)** The RT-LAMP reaction was performed using a portable thermostatic device at 65°C for 30 min. **(C)** Detection the amplified products by SYBR Green I and LFD. The 5′ ends of the BIP and LB primers were labeled with biotin and FAM, respectively, facilitating detection by the LFD. The labeled amplification product of SMV-SC7 will be coupled with Au-NP-anti-biotin and anti-6-FAM antibodies to produce a signal on the detection line, which indicates a positive result.

Therefore, a simple and rapid RNA extraction method using MBs was optimized, a set of LAMP primers based on the P3 gene sequence of SMV-SC7 was designed, and integration with an LFD for sequence-specific detection of the SMV-SC7 strain was achieved (working principle is illustrated in Figure 1). The LAMP assays were used to assess primer sensitivity and specificity. Furthermore, 194 soybean leaves collected from fields were utilized to assess the performance of this detection platform for SMV-SC7. The visual results were semi-quantified through gray-scale analysis and normalization. The quantitative analysis was subsequently performed to determine SMV-SC7 viral load in these field samples. In conclusion, a rapid, portable, cost-effective, and precise platform was established to detect SMV-SC7, aimed at preventing soybean yield reduction.

## 2 Materials and methods

### 2.1 Sample collection

SMV-SC3, SMV-SC7, and SMV-SC15 strains were supplied by the National Center for Soybean Improvement (NCSI) at Nanjing Agricultural University, Nanjing, China, and kept in the leaves of soybean cultivar Nannong 1138-2. In 2023, 194 soybean cultivars (see Supplementary Table 1) were cultivated at the Shenfeng Experimental Station in the Taigu district—a part of College of Agriculture, Shanxi Agricultural University. During the seedling phase, 3–4 leaves were picked and subsequently stored at −80°C.

### 2.2 RNA extraction, optimization, and cDNA synthesis

MBs size, incubation time, number of washes and bead-to-sample mass ratio affected RNA extraction efficiency by affecting RNA binding efficiency. Therefore, the extraction protocol was optimized based on the MBs method for nucleic acid extraction (Wang et al., 2015). Parameters, such as MBs size (40, 200, 300, and 400 nm), incubation time (1, 3, 5, 7, 9, and 11 min), number of washes (2–4 times), and bead-to-sample mass ratio (1:500, 1:1000, 1:1500, 1:2000, and 1:2500) were optimized respectively during RNA extraction using MBs while other parameters were maintained under constant conditions. Total RNA was isolated from soybean leaves according to the optimized protocol, which is described as follows: (1) A specific mass of soybean leaves was added to a 1.5 mL enzyme-free centrifuge tube; (2) After the addition of lysis buffer, the mixture was incubated at room temperature for 3 min, then centrifuged at 13,000 rpm for 3 min; (3) The MBs and supernatant were thoroughly mixed by vortexing in a new 1.5 mL nuclease-free centrifuge tube; (4) After being vortexed, the mixture was incubated at room temperature for 3 min; (5) The tube was placed on a magnetic rack for 15 s until the solution clarified; (6) Freshly prepared 80% ethanol was added and the supernatant was removed after 15 s incubation; (7) The MBs were air-dried and nuclease-free H_2_O was added.

To compare this method with the kit extraction method, the EZ-10 DNA away RNA Mini-Preps Kit (Sangon Biotech, Shanghai, China) was used also used to isolate total RNA from soybean leaves.

The cDNA synthesis was carried out using the GoScript™ Reverse Transcription System (Promega, USA).

### 2.3 LAMP primer designing and synthesis

The P3 gene is a highly conserved gene, which plays a crucial role in virus movement and replication, as well as host infection (Luan et al., 2019). After comparing the P3 genomic sequences of SMV-SC3, SMV-SC4, SMV-SC6, SMV-SC7, and SMV- SC15 received from GenBank (MH919385.1, MN539670.1, KP710867.1, MH919384.1, MH919386.1), Primer Explorer V5 was used to design a set of LAMP-specific primers for SMV-SC7 (Supplementary Table 1). The 5′ ends of SC7-BIP and SC7-LB primers were labeled with biotin and FAM, respectively, for visualization using LFD. The primers were synthesized by Sangon Biotech, Ltd. (Shanghai, China).

### 2.4 RT-LAMP assay optimization

The RT-LAMP reaction system for SMV-SC7 was optimized and a real-time fluorescence method was employed for quantification. The assay optimization involved five parameters: temperature (62.6, 65.0, 67.0, 68.1, and 68.6°C), Bst DNA polymerase concentration (2, 4, 6, 8, and 10 U), inner to outer primer ratio (1:1, 2:1, 4:1, 6:1, and 8:1), Mg^2+^ concentration (3–7 mM), and betaine concentration (0.4, 0.6, 0.8, 1.0, and 1.2 M). Each parameter was repeated three times.

### 2.5 RT-LAMP-LFD assay

The RT-LAMP reaction mixture consisted of 2.0 μL 10× Isothermal Amplification Buffer, 4.0 mM MgSO_4_, 1.2 μM inner primers, 0.2 μM outer primers, 0.6 μM SC7-LB, 0.8 mM dNTPs (Solarbio, Beijing), 1.0 M betaine (Solarbio, Beijing), 4.0 U Bst DNA polymerase (New England Biolabs), and a cDNA template (100 ng/μL), with sterilized ddH_2_O added to adjust the total volume to 20 μL. The reaction was incubated at 65°C for 30 min. The RT-LAMP amplification products were checked by 2% agarose gel electrophoresis.

All components and procedures used in the RT-LAMP-LFD reaction were the same as used in the RT-LAMP reaction, except biotinylated inner primers (SC7-BIP-Bio) and a fluorescently labeled loop primer (SC7-LB-FAM).

### 2.6 RT-LAMP product analysis

For the colorimetric RT-LAMP reaction, 2.0 μL of SYBR Green I at a 1000× concentration (Solarbio, Beijing) was added to 9 μL of the RT-LAMP amplification product. The results were determined by observing the color shift—an orange color indicating a negative reaction and a green color signifying a positive reaction (Iwamoto et al., 2003; Wu et al., 2024).

For the RT-LAMP-LFD reaction, 20 μL of the RT-LAMP product was added to 70 μL of sterilized ddH_2_O. The LFD strips were immersed in the solution and incubated for 2 min, and then removed and photographed. When test and control lines appeared simultaneously (Figure 1), the result was considered positive; when only a control line appeared, the result was considered negative. If only the test line appeared, the result was deemed doubtful and required a retest. ImageJ software was utilized to extract gray-scale data from the test lines, and the signals were subsequently normalized (Mao et al., 2024).

### 2.7 Specificity and sensitivity evaluation

The specificity of RT-LAMP, RT-PCR, and RT-qPCR reactions for SMV-SC7 was assessed utilizing SMV-SC3, SMV-SC7, and SMV-SC15 cDNA templates.

The cDNA of SMV-SC7 was subjected to a 10-fold serial dilution, for achieving a concentration range from 10^1^ to 10^−5^ ng/μL to evaluate the sensitivity of RT-LAMP, RT-qPCR and conventional PCR. Sterilized ddH_2_O, heated to 121°C, served as a negative control. The fluorescence signal was detected in real time by adding SYBR Green I, and then the limit of detection (LOD) of RT-LAMP was determined. The RT-PCR results were verified by agarose gel electrophoresis. Each reaction was conducted in triplicate. The copy number calculations were made according to the formula (Nakiboneka et al., 2024):


(1) $$\text{Copy/}\mu \text{L}=\frac{\text{Amount}\left(\text{ng/}\mu \text{L}\right)\times {\text{Avogadro}}^{\text{'}}\text{s constant}}{\text{Length}\left(\text{bp}\right)\times \text{1}.\text{00E + 09}\times \text{660}}$$

### 2.8 RT-PCR

The RT-PCR reaction mixture consisted of 0.3 μL of 10 × EasyTaq^®^ Buffer, 0.2 μM SMV-SC7-F (5′-AAAAGGGGTGGAGTTGTG-3′) and SMV-SC7-R (5′-CTTGTATGACGGTGGTACT-3′), respectively, 0.2 mM dNTPs, 2.5 U EasyTaq^®^ DNA Polymerase (TransGen Biotech), and 1 μL 100 ng/μL cDNA template, with sterilized ddH_2_O added to adjust the total volume to 15 μL. The following settings were used for the RT-PCR reaction: an initial denaturation at 94°C for 2 min, followed by 32 cycles of denaturation at 94°C for 30 s, annealing at 60°C for 30 s, and extension at 72°C for 20 s, and then final extension at 72°C for 5 min. The RT-PCR amplification products were analyzed by 1% agarose gel electrophoresis.

### 2.9 RT-qPCR

To evaluate the feasibility of detecting SMV-SC7 in field samples, RT-qPCR amplification was performed by using the Bio-RAD CFX96 Touch instrument. The amplification mixture for RT-qPCR included 10 μL 2X TransStart Tip Green qPCR SuperMix (TransGen Biotech, Beijing), 0.2 μM SMV-SC7-F (5′-AAAAGGGGTGGAGTTGTG-3′) and SMV-SC7-R (5′-CTTGTATGACGGTGGTACT-3′), respectively, and 1 μL 100 ng/μL cDNA template, with sterilized ddH_2_O added to adjust the total volume to 20 μL. The following settings were used for the RT-qPCR reaction: an initial denaturation at 94°C for 30 s, followed by 37 cycles of denaturation at 94°C for 5 s and extension at 61°C for 30 s. Finally, a quantitative standard curve for SMV-SC7 was established using a 10-fold serial dilution of SMV-SC7 cDNA to analyze the viral load.

### 2.10 Field sample testing

To conduct a direct field screening of soybean varieties with resistance to SMV-SC7, the leaves of 194 soybean varieties were gathered at the seedling stage and analyzed using colorimetric RT-LAMP, RT-LAMP-LFD, and RT-qPCR methods. Accuracy = (TP+TN)/(TP+TN+FP+FN); Sensitivity = TP/(TP+FN); Specificity = TN/(FP+TN). TP, True Positive; FN, False Negative; TN, True Negative; FP, False Positive.

## 3 Results

### 3.1 RT-LAMP primers designed to detect of SMV-SC7

SMV—a member of the genus *Potyvirus* within the family Potyviridae—possesses a single-stranded, positive-sense ~9.6 kb long RNA. It encodes at least 11 proteins, including potyvirus 1 (P1), helper-component proteinase (HC-Pro), and potyvirus 3 (P3), and so forth (Urcuqui-Inchima et al., 2001). Among them, the P3 gene is closely related to replication, movement, and pathogenesis in SMV (Luan et al., 2016). Therefore, LAMP primers were designed to target a specific segment of the P3 gene of SMV-SC7. Five primers set were comprised of a pair outer primer (SC7-F3/SC7-B3), a pair inner primer (SC7-FIP/SC7-BIP), and a loop primer (SC7-LB). The primer design schematic diagram and primer sequences were shown in Supplementary Figure 1 and Supplementary Table 1, respectively. Biotin and FAM were labeled at the 5' ends of SC7-BIP and SC7-LB, respectively, for lateral flow strip detection (Supplementary Table 1).

### 3.2 Specificity evaluation of RT-LAMP assay for SMV-SC7

**Figure 2:**
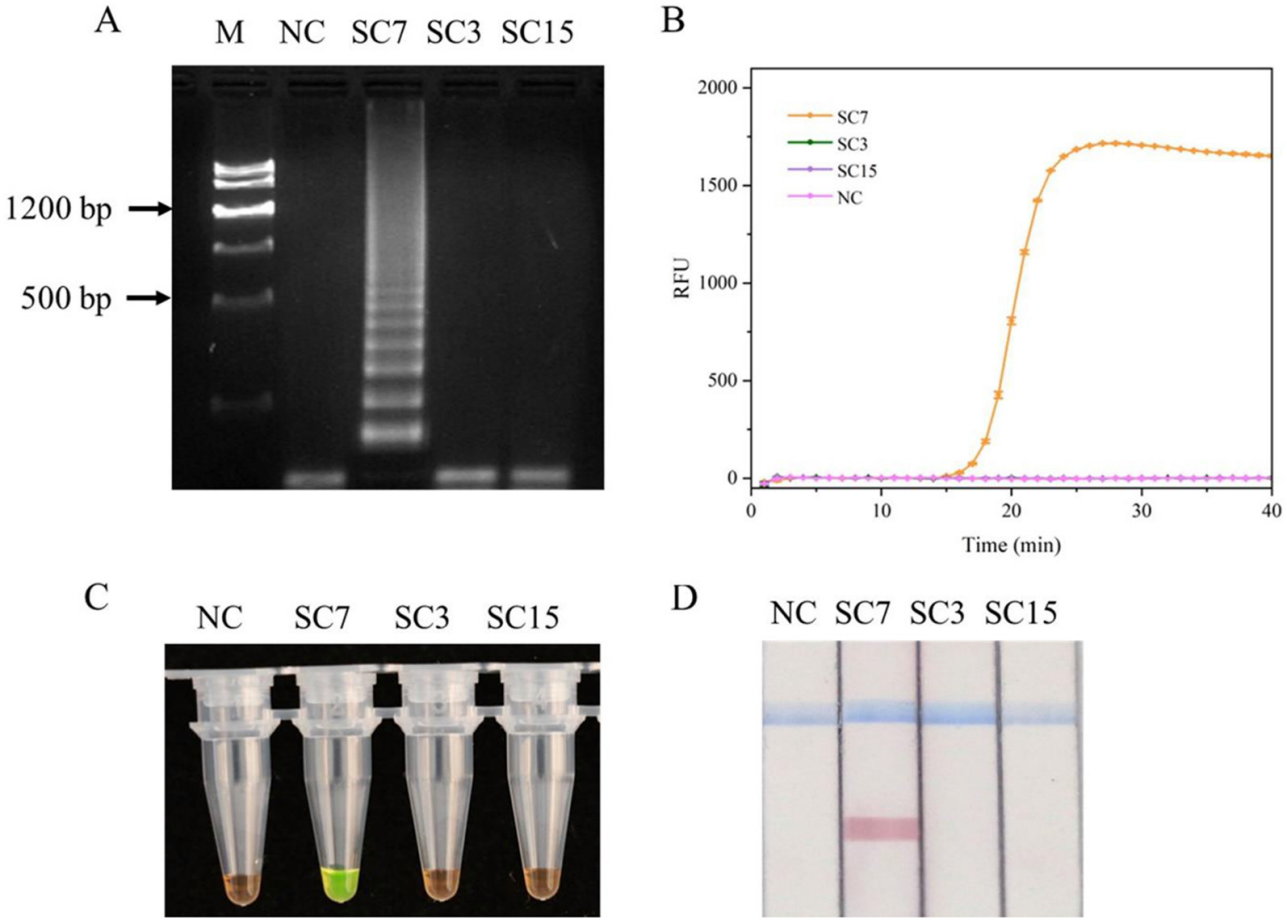
Evaluation of the RT-LAMP assay specificity for SMV-SC7. The cDNA of SMV-SC3, SMV-SC7, and SMV-SC15 were used to assess the RT-LAMP assay's specificity. **(A)** Gel electrophoresis image of LAMP reaction products. **(B)** Evaluation of specificity in real-time RT-LAMP. **(C)** Assessment of specificity in colorimetric RT-LAMP stained with SYBR Green I. **(D)** Specificity was assessed using LFD in RT-LAMP-LFD. M, DNA Marker III; NC, negative control.

NCBI has only published the sequences of five SMV strains (SC3, SC4, SC6, SC7, and SC15). The sequence of SMV-SC3, SMV-SC4, and SMV-SC6 are completely identical within the region relevant to primer design. To evaluate the specificity of the designed primers to amplify SMV-SC7, RT-LAMP experiments were conducted by using SMV-SC7, SMV-SC3, and SMV-SC15 cDNA. The J-shaped curve and distinctive ladder-like pattern were observed in SMV-SC7 only (Figures 2A, B). For visual detection of LAMP products, SYBR Green I and LFD were employed, respectively, with outcomes consistent with those from real-time fluorescence quantification and gel electrophoresis analysis (Figures 2C, D). In addition, RT-PCR and RT-qPCR specificity were efficiently evaluated. As shown in Supplementary Figure 2, RT-PCR and RT-qPCR could specifically amplify SMV-SC7.

### 3.3 RT-LAMP reaction optimization

**Figure 3:**
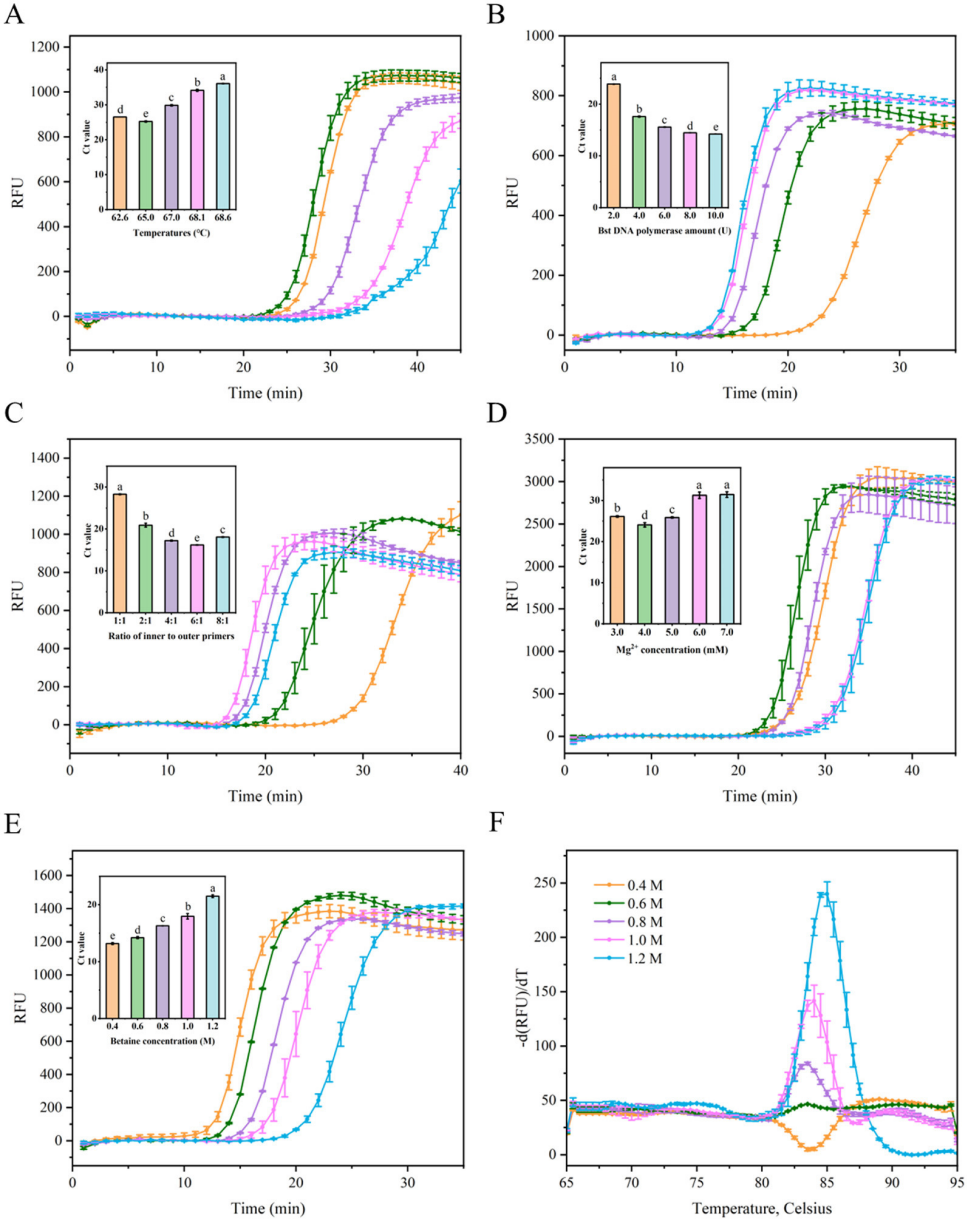
Optimization the parameters of the LAMP reaction for detecting SMV-SC7. **(A)** Evaluation fluorescence signals and Ct value in the RT-LAMP reaction system with different temperatures (68.6, 68.1, 67.0, 65.0, and 62.6°C). **(B)** Examination fluorescence signals and Ct value in the RT-LAMP reaction system with different amount of Bst DNA polymerase (2, 4, 6, 8, and 10 U). **(C)** Assessment fluorescence signals and Ct value in the RT-LAMP reaction system with different ratio of inner to outer primers (1:1, 2:1, 4:1, 6:1, and 8:1). **(D)** Evaluation fluorescence signals and Ct value in the RT-LAMP reaction system with different Mg^2+^ concentration (3, 4, 5, 6, and 7 mM). **(E)** Assessment fluorescence signals and Ct value in the RT-LAMP reaction system with different betaine concentration (0.4, 0.6, 0.8, 1.0, and 1.2 M). **(F)** Examination melting curve in the RT-LAMP reaction system with different betaine concentration (0.4, 0.6, 0.8, 1.0, and 1.2 M). Error bars represent SD of mean, *n* = 3. *P* < 0.05 was considered statistically significant. RFU, relative fluorescence units.

Five factors were assessed to optimize the LAMP tests, including the reaction temperature, concentration of the Bst DNA polymerase, the ratio of inner to outer primers, Mg^2+^ concentration, and betaine concentration. As shown in Figure 3A, the cycle threshold (Ct) of the LAMP reaction at 65°C was ~25.2, while the Ct values at other temperatures ranged from 26.5 to 36.1, which were higher than the Ct value at 65°C, indicating that optimal amplification efficiency was achieved at 65°C with the lowest Ct value. Excessively high or low temperatures resulted in lower amplification efficiencies compared to the moderate temperature. The Ct value decreased by 26.3% as the amount of Bst DNA polymerase was increased from 2 U to 4 U. However, when the Bst DNA polymerase amount ranged from 4 U to 10 U, the Ct value did not show a substantial further decrease (Figure 3B). Considering cost-effectiveness, 4 U of Bst DNA polymerase were selected as the optimal concentration. As shown in Figure 3C, the Ct value of the LAMP reaction with an inner-to-outer primer ratio of 6:1 was ~16.2, which was lower than the Ct values (~17.2–28.3) for other inner-to-outer primer ratios (1:1, 2:1, 4:1, and 8:1). Thus, the optimal inner-to-outer primer ratio in the RT-LAMP system was determined to be 6:1. Furthermore, an improvement (reduction) in the LAMP Ct value was observed at an Mg^2+^ concentration of 4.0 mM, with decreases of 8.0%−23.5% compared to other Mg^2+^ concentrations (Figure 3D). This indicated that optimal amplification efficiency in this LAMP system was achieved at a 4.0 mM Mg^2+^ concentration, yielding the lowest Ct value. The Ct values of LAMP reaction decreased with betaine concentrations ranging from 1.2 to 0.4 M (Figure 3E). However, the dissolution curve peaks were not sufficiently sharp at betaine concentrations of 0.4, 0.6, and 0.8 M, indicating greater instability in the reaction system (Figure 3F). Consequently, a final concentration of 1.0 M betaine was chosen based on the combination of amplification efficiency and system stability.

In summary, the optimal reaction parameters were established as follows: 4.0 U Bst DNA polymerase, inner to outer primer ratio-6:1, 4.0 mM Mg^2+^, and 1.0 M betaine, within a 20 μL reaction system maintained at 65°C.

### 3.4 MB-based RNA extraction optimization

**Figure 4:**
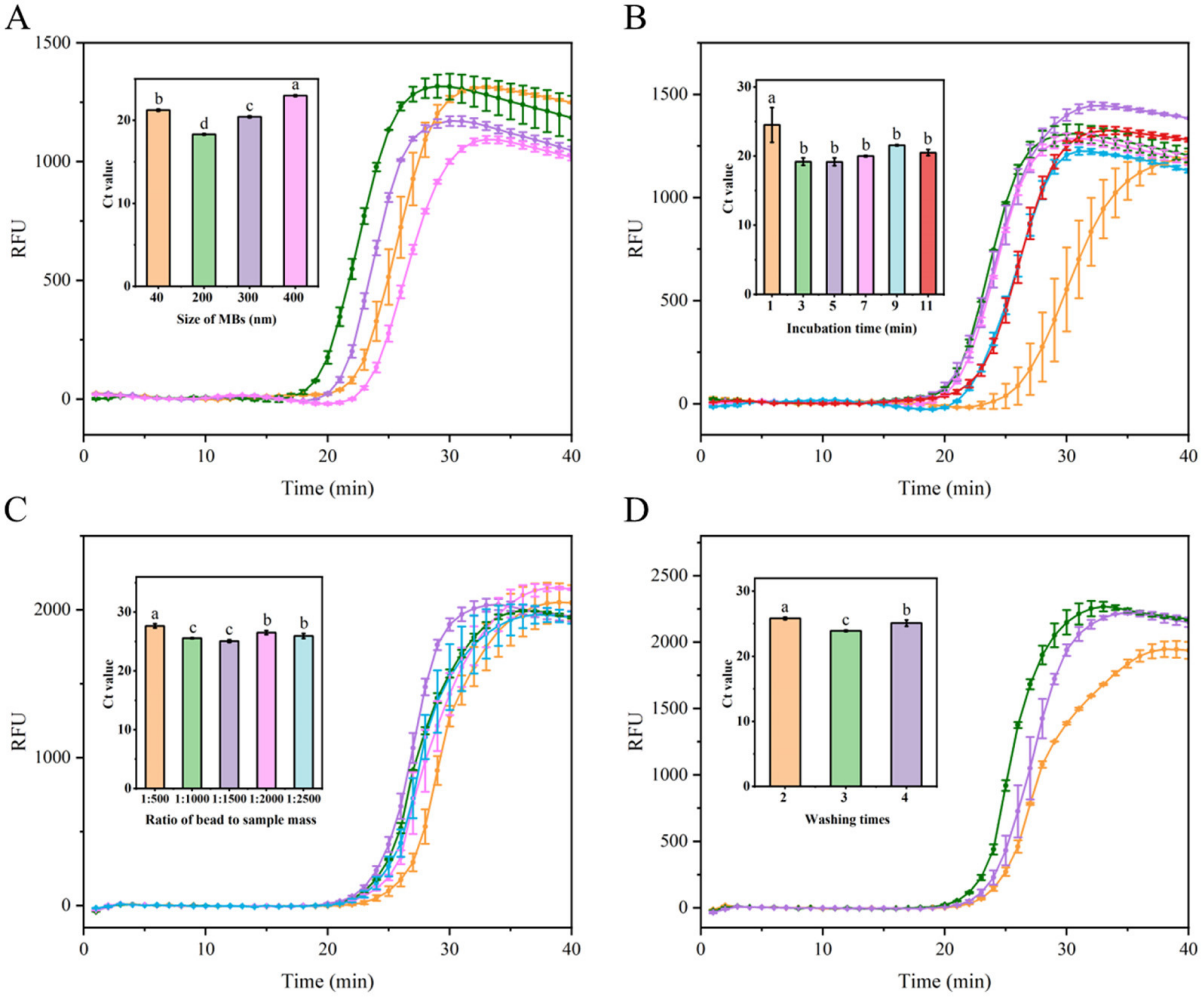
Optimization of nucleic acid extraction utilizing MBs. **(A)** Evaluation fluorescence signals and Ct value in the RT-LAMP reaction system with different Mbs sizes (40, 200, 300, and 400 nm). **(B)** Assessment fluorescence signals and Ct value in the RT-LAMP reaction system with different incubation time (1, 3, 5, 7, 9, and 11 min). **(C)** Examination fluorescence signals and Ct value in the RT-LAMP reaction system with different ratio of MBs to sample mass at 1:500, 1:1000, 1:1500, 1:2000, and 1:2500. **(D)** Evaluation fluorescence signals and Ct value in the RT-LAMP reaction system with different washes times (2, 3, and 4 times). Error bars represent SD of mean, *n* = 3. *P* < 0.05 was considered statistically significant. RFU, relative fluorescence units.

To enhance extraction efficiency and obtain high-quality RNA from soybean leaves, an MB-based RNA extraction process was optimized. Initially, the influence of MB size on nucleic acid extraction efficiency was investigated. As depicted in Figure 4A, 200 nm MBs demonstrated optimal performance, characterized by the shortest amplification time and lowest Ct value. Conversely, 300 and 400 nm MBs showed lower performances, potentially due to their larger particle size and comparatively smaller specific surface area, suggesting a reduced capacity for RNA binding. For 40 nm MBs, diminished performance was observed, which can be attributed to the lowest amount of SiO_2_ coating on MBs (He et al., 2021), consequently reducing their RNA capture capacity. Subsequently, to ensure adequate MB binding to RNA, sufficient incubation time was found to be critical. Extraction efficiency improved with incubation time increased from 1 to 3 min. However, extending the incubation time beyond 3 min did not further enhance amplification efficiency and instead slightly decreased the efficiency, possibly due to RNA degradation from prolonged incubation time (Figure 4B). Thus, a 3-min incubation time resulted in optimal performance, characterized by the shortest amplification duration. Consequently, the optimal incubation time for the MBs to capture free viral RNA was determined to be 3 min. Given that the amount of MBs affects RNA extraction efficiency, the MB to sample mass ratio was investigated. As shown in Figure 4C, performance improved as the sample input increased within a ratio range of 1:500–1:1500. However, when the ratios exceeded 1:1500, the amplification efficiency ceased to increase and rather slightly decreased. This reduction may be due to fewer numbers of MBs, resulting in inadequate MB binding to the nucleic acid, thereby diminishing extraction efficiency. Additionally, the impact of different washing times (using 80% ethanol) on extraction efficiency was examined. Among all the washing times, three times washing provided the best performance, achieving the shortest amplification time, whereas two and four times washing resulted in lower performances (Figure 4D). This may be attributed to the point that insufficient washing may leave residues of impurities and inhibitors, thereby affecting downstream processing efficiency. Conversely, excessive washing may lead to the loss of nucleic acids bound to the MBs, thereby reducing amplification efficiency.

In summary, the optimal conditions for RNA extraction using MBs were established as follows: 200 nm beads, a 3-min incubation period, a 1:1500 bead-to-sample ratio, and three washes with 80% ethanol.

### 3.5 Evaluation of MB-based RNA extraction method with RT-LAMP

To evaluate the sensitivity of RT-LAMP, RT-qPCR, and RT-PCR for SMV-SC7 detection, the cDNA was diluted in a 10-fold gradient. As shown in Supplementary Figure 3D, SMV-SC7 was successfully detected by real-time fluorescent RT-LAMP, ranging from 10^1^ to 10^−4^ ng/μL, with an LOD of 10^−4^ ng/μL (24 copies/μL). The J-shaped curve was observed by RT-qPCR, ranging from 10^1^ to 10^−4^ ng/μL (Supplementary Figure 3A). There was a good linear relationship between cDNA concentration and cycle threshold time (CT) (Supplementary Figure 3B). However, RT-PCR could only detect cDNA concentrations up to 10^−2^ ng/μL (2.4 × 10^3^ copies/μL) (Supplementary Figure 3C).

**Figure 5:**
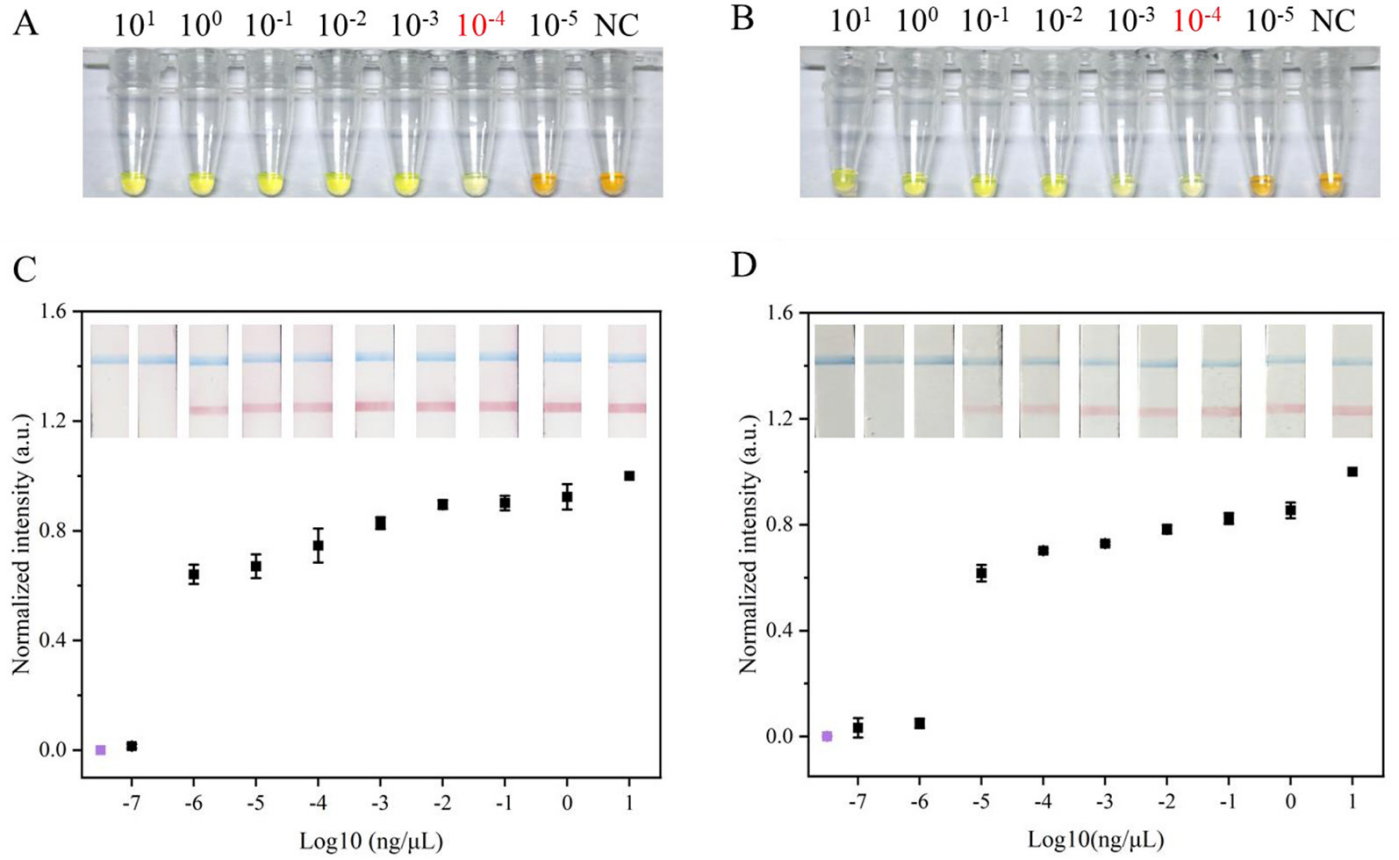
Evaluation of RNA extraction based on MBs the method and the commercial RNA extraction kit method. Sensitivity evaluation of **(A)** commercial RNA extraction kit method and **(B)** the MBs based RNA extraction method used colorimetric LAMP visual detection, respectively. Orange color indicates negative, and a green color signifies positive. Sensitivity evaluation of **(C)** commercial RNA extraction kit method and **(D)** the MBs based RNA extraction method used LFD visual detection, respectively. When control line (blue) and test line (red) appeared simultaneously, the result was considered positive; when only a control line appeared, the result was considered negative. Visual signals from the LFD were quantified using ImageJ, and subsequent normalization was carried out. The y-axis **(C,D)** represents the normalized intensity of the gray-scale analysis results and the x-axis **(C,D)** denotes the logarithm of SC7 template concentrations. The concentrations 10^1^-10^−7^ ng/μL represent different concentrations of SC7 template used for testing purposes; NC refers to negative control. Negative control by RT-LAMP-LFD was colored purple. Error bars represent SD of mean, *n* = 3.

To assess the efficacy of the MB-based RNA extraction method from soybean leaves, its performance was compared with a commercial RNA extraction kit using RT-LAMP. The real-time fluorescence results showed that both MB-based method and commercial kit successfully detected SMV-SC7, with similar amplification times. Notably, the Ct value for the commercial kit was significantly lower than that of the MB-based method (Supplementary Figure 4). Subsequently, colorimetric RT-LAMP assays were conducted to evaluate the efficiency of the two RNA extraction methods using serially diluted SMV-SC7 cDNA, ranging from 10^1^ to 10^−5^ ng/μL. As depicted in Figures 5A, B, for both RNA extraction methods, the LAMP reaction solution turned green at SMV-SC7 cDNA concentrations ranging from 10^−4^ to 10^1^ ng/μL. However, at concentrations lower than 10^−4^ ng/μL (24 copies/μL), the test solutions stayed orange, matching in color with that of the negative control. These findings demonstrated that the two RNA extraction methods did not impact the sensitivity of LAMP visualization. Finally, RT-LAMP-LFD assays using serially diluted SMV-SC7 cDNA were performed to assess the sensitivity of the MB-based RNA extraction method. As shown in Figures 5C, D, the LFD showed that with the commercial RNA extraction kit, a red test band was visible at cDNA concentrations from 10^−6^ to 10^1^ ng/μL, whereas the MB-based method could only detect cDNA concentrations up to 10^−5^ ng/μL (2.4 copies/μL). These findings suggest that the RT-LAMP-LFD approach is more sensitive than colorimetric RT-LAMP for both RNA extraction methods, demonstrating that incorporating an LFD into the LAMP assay enhances sensitivity.

As LFD visualization is limited to qualitative analysis, the test lines on the LFD are subjected to the gray-scale analysis using ImageJ for semi-quantitative assessment. The results demonstrated consistency between the gray-scale analysis findings and visualization outcomes (Figures 5C, D).

### 3.6 Field soybean sample testing using RT-LAMP-LFD assay

To assess the accuracy and reliability of the RT-LAMP-LFD assay for SMV-SC7 in field applications, the SMV-SC7 infection status was evaluated on leaves from 194 soybean varieties at the Shanxi Shenfeng Experimental Field. Initially, RNA from the leaves of 194 soybean varieties were extracted using the MB-based RNA extraction method. Given that RT-qPCR is regarded as the gold standard in laboratories and clinics to diagnose multiple diseases (Yi et al., 2024), RT-qPCR testing was conducted on 194 field samples, using a threshold of 37 cycles to determine positivity. Subsequently, colorimetric RT-LAMP and RT-LAMP-LFD methods were employed to detect SMV-SC7. The results showed that 62 samples were identified as positive for SMV-SC7 by RT-qPCR (Ct values of 21.3–36.8), whereas 41 samples were validated as positive by colorimetric LAMP, and 59 samples were identified as positive by RT-LAMP-LFD. The SMV-SC7 positive rate was 32.96% (62/194), 21.12% (41/194), and 30.41% (59/194) with RT-qPCR, colorimetric LAMP, and RT-LAMP-LFD, respectively. Compared with the RT-qPCR method, the colorimetric RT-LAMP and RT-LAMP-LFD assays, respectively, demonstrated a specificity of 97.73% (129/132) and 100% (132/132), a sensitivity of 61.29% (38/62) and 95.16% (59/62), and an overall accuracy of 86.08% (167/194) and 98.45% (191/194) (Table 1, Supplementary Table 2, Supplementary Figures 5, 6). These findings suggested that the RT-LAMP-LFD assay demonstrated an excellent diagnostic agreement with the RT-qPCR assay for SMV-SC7 detection and could be a useful tool to qualitatively analyze endpoint results in field soybean samples.

**Table 1:** Field samples evaluated by the colorimetric RT-LAMP and the RT-LAMP-LFD.

**Total specimens tested**	**Sensitivity**	**Specificity**	**Accuracy**	**Total samples**
Colorimetric RT-LAMP	38/62 (61.29%)	129/132 (97.73%)	167/194 (86.08%)	194
RT-LAMP-LFD	59/62 (95.16%)	132/132 (100%)	191/194 (98.45%)	194

**Figure 6:**
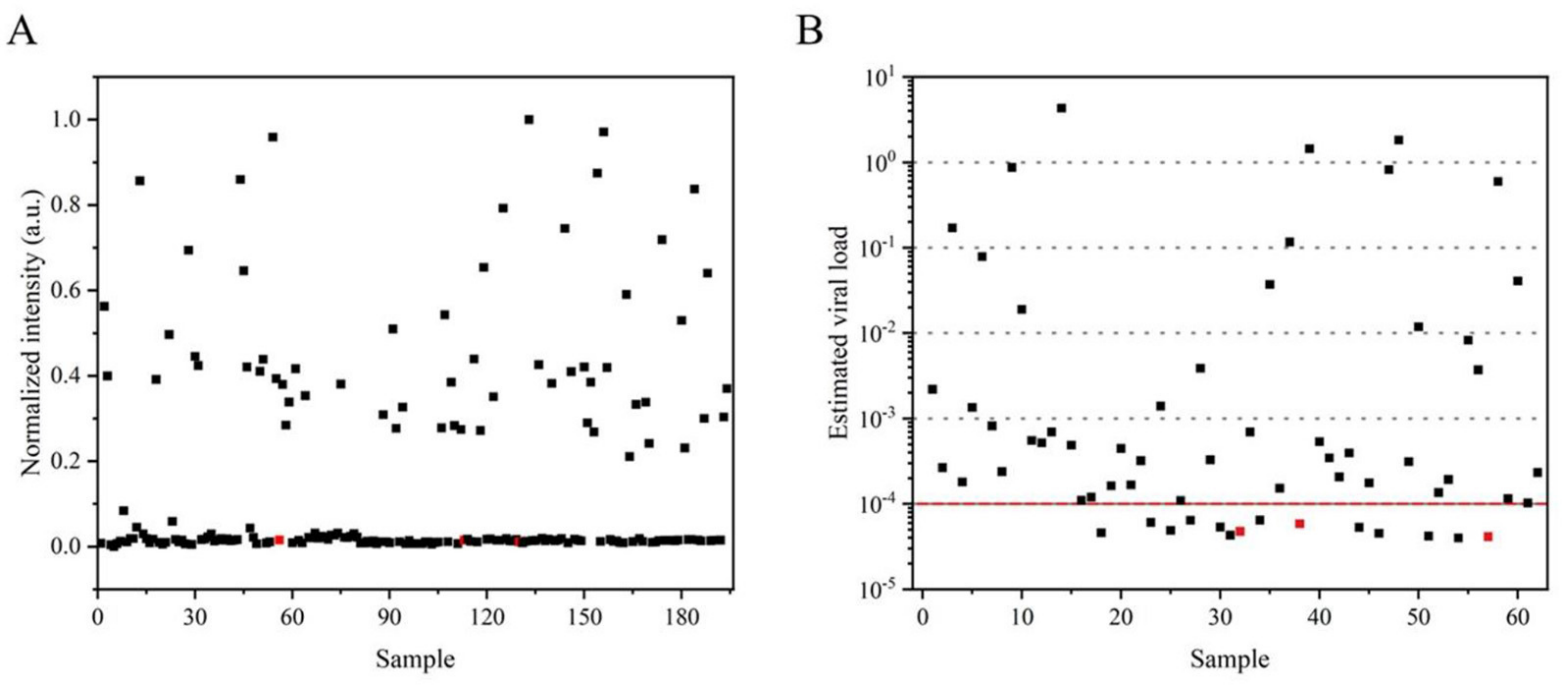
Application of the detection platform in field samples. **(A)** Visual signals from the RT-LAMP-LFD were quantified using ImageJ, with subsequent normalization applied to field samples. **(B)** The estimation of viral load in each sample was determined using the quantitative standard curve obtained from RT-qPCR. Samples identified as negative by RT-LAMP-LFD are marked in red.

Given the qualitative limitations of RT-LAMP-LFD visualization at the endpoint, the visual signals were quantified using ImageJ, and normalization was applied to the field samples (Figure 6A). Color data extracted by ImageJ confirmed consistency with the results described earlier, although this was not a fully quantitative analysis. Consequently, a 10-fold gradient dilution of the SMV-SC7 cDNA template was performed for the RT-qPCR assay to establish a linear correlation between Ct values and viral load, facilitating the estimation of SMV-SC7 load in field soybean samples (Supplementary Figures 3A, B). The median estimated viral load in positive samples was 10^−3.5^ ng (2.4 × 10^1.5^ copies), with a range from 10^0.6^ to 10^−4.4^ ng/μL (Figure 6B). All false negatives in the RT-LAMP-LFD assay exhibited estimated viral loads of <10^−4^ ng (24 copies), likely falling below the threshold required for viral transmission (Larremore et al., 2021). However, of the 14 samples that fell below this threshold, the RT-LAMP-LFD still detected SMV-SC7 in more than half of them. At levels above this threshold, the assay achieved 100% sensitivity. In addition, viral load variations among different varieties indicated that these varieties had different resistance levels to SMV-SC7. These findings suggest that the assay platform could potentially serve as a quantitative tool to analyze field soybean samples. The RT-LAMP-LFD visualization platform developed in this study provides rapid, portable, cost-effective, and precise detection of SMV-SC7 in soybeans, offering a new strategy for monitoring SMV-SC7 infection.

## 4 Discussion

Soybean mosaic disease—commonly attributed to SMV—is a prevalent viral disease affecting soybeans, impacting seed yield and quality (Hajimorad et al., 2018; Liu et al., 2016; Usovsky et al., 2022). Point-of-care testing (POCT) is critical for diagnosing and managing soybean mosaic disease in various settings, particularly in remote places. The traditional approach for detecting and diagnosing viruses that infect crops is to examine their biological properties such as symptoms (Debreczeni et al., 2011). However, these tests may take several weeks. PCR-based methods are cumbersome because they require the initial isolation of nucleic acids, followed by amplification of the target sequence and analysis of the products on an agarose gel (Bhat et al., 2022). By contrast, LAMP can be performed at a single temperature without the need for thermal cyclers, and the results can be visualized as a color change and LFD (Notomi et al., 2000; Chen et al., 2023). In this study, a simple and rapid MB-based RNA extraction approach was optimized and combined with RT-LAMP-LFD for specific detection of the SMV-SC7 strain. The RT-LAMP-LFD platform did not require specialized equipment or technical expertise, facilitating early diagnosis of plant diseases, such as SMV-SC7. This enables timely interventions to control outbreaks, mitigate the impact of SMV-SC7 on soybean yields, and minimize economic losses for farmers.

In recent years, MB-based nucleic acid extraction methods have been employed across various fields (Faggi et al., 2005; Faye et al., 2024; Klein et al., 2020; Wang et al., 2024). Typically, the MB-based RNA extraction process involves stages of lysis, incubation, washing, and elution. Research has indicated that the general yield of nucleic acids from MB-based methods ranges from 70% to 80%. The primary cause of this loss has been attributed to the elution step (Branch et al., 2017; Wielinga et al., 2011; Xu et al., 2017; Zhang et al., 2015; Zhang Z. M. et al., 2022). Consequently, in this study, RNA was not eluted from the MBs during extraction but was rather used directly for RT-LAMP.

In addition, in animal nucleic acid extraction, the MB-based method can eliminate the need for centrifugation (Klein et al., 2020; Zhang et al., 2015; Zhang Z. M. et al., 2022). However, due to the rigid polysaccharide cell walls of plant cells, centrifugation is still required during the extraction of nucleic acids from plant tissues. Thus, developing techniques to eliminate centrifugation and further simplify the extraction process remains a crucial area for future research in plant tissue nucleic acid extraction.

Methods for visualizing RT-LAMP reaction results include pH-sensitive dyes (Luo et al., 2021), metal ion indicators (Yang et al., 2024), nanoparticles (Wang et al., 2023), and fluorescent dyes (Wu et al., 2024), among others. In the present study, SYBR Green I and LFD were utilized for RT-LAMP assay visualization. In colorimetric RT-LAMP detection, sensitivity can reach 10^−4^ ng/μL (24 copies/μL), whereas RT-LAMP-LFD methods can achieve sensitivities up to 10^−5^ ng/μL (2.4 copies/μL). Nimitphak et al. developed a LAMP-LFD assay for the detection of shrimp hepatopancreatic parvovirus (PmDNV), and found that the integration of LAMP with LFD could enhance the sensitivity of traditional RT-LAMP assays, which is consistent with the result of our study. And the sensitivity of the developed assay showed an improvement of one and three orders of magnitude, respectively, compared with RT-qPCR and RT-PCR assays (Supplementary Figure 3). Since RT-qPCR is considered the gold standard for diagnosing diseases in laboratories and clinics (Yi et al., 2024), the viral load in infected soybeans was assessed by RT-qPCR in our study. The results showed that the viral load of all infected soybeans was higher than 10^−5^ ng (2.4 copies) (Figure 6A). Therefore, a detection limit of 10^−5^ ng (2.4 copies) is sufficient for field applications.

The RT-LAMP-LFD results can be visually recognized with the naked eye. While visualization detection enables qualitative analysis of endpoints by identifying the presence or absence of test lines, it is not suitable for absolute quantitative analysis due to the “plateau effect” observed in RT-LAMP product pre-amplification. Given the limitations of qualitative analysis in visually inspecting RT-LAMP-LFD endpoints, the visual signals from RT-LAMP-LFD were quantified through color data extraction using ImageJ and subsequent normalization of the samples. However, this approach provides semi-quantitative analysis only. Finally, RT-qPCR was employed to establish a linear relationship between Ct values and viral load, enabling the assessment of SMV-SC7 load in the field samples. This suggests that the assay platform has the potential to function as a quantitative analysis tool.

Screening of SMV-resistant soybean germplasms directly in the fields is a primary strategy for breeders. In this study, RT-LAMP primers were specifically designed for detecting the SMV-SC7 strain. The colorimetric RT-LAMP and RT-LAMP-LFD assays were employed to evaluate SMV-SC7 infection in leaves of 194 soybean cultivars, selected at the seedling stage from the fields that had been continuously cultivated with soybeans for 6 years. Consequently, 132 of these cultivars showed no signs of SMV-SC7 infection, suggesting their potential resistance to this strain and highlighting their suitability as candidates for future breeding programs aimed at enhancing SMV resistance. In contrast (Li et al., 2024) found that only 69 out of 200 soybean varieties were resistant to SMV-SC15 at the seedling stage, under similar conditions of continuous cultivation. This indicates that SMV-SC15 exhibits greater toxicity and dominance than SMV-SC7, aligning with findings reported by Zhang K. et al. (2022).

The RT-LAMP-LFD assay consists of Mb extraction RNA, LAMP amplification and product analysis by LFD. The MB-based method is more economical, and each sample is ~1.2 dollars inexpensive than commercial kits (Palani et al., 2019; Simões et al., 2013). RT-LAMP is an isothermal-based amplification assay that does not require complex laboratory equipment (Notomi et al., 2000). Bst DNA polymerase used for LAMP amplification has strong strand displacement activity and high temperature tolerance, which is particularly important for field applications in resource-limited environments. And the use of the LFD makes it possible to visualize to analyze the products. Furthermore, the total RT-LAMP-LFD analysis procedure can be completed within ~75 min including MB-based RNA extraction (15 min), reverse transcription (30 min), RT-LAMP amplification (30 min) and result interpretation (<2 min). However, the total RT-qPCR analysis procedure can be completed within ~140 min including commercial kit RNA extraction (30 min), reverse transcription (30 min) and RT-qPCR reaction (80 min). RT-LAMP-LFD reduced the time by ~46% compared to RT-qPCR. In conclusion, the assay enables rapid and portable detection of SMV-SC7 and providing technical support to identify SMV-SC7-infected soybeans.

This study has several limitations. First, the criteria used to test individuals may influence group characteristics, potentially leading to deviations in our results. Second, although the MB-based RNA extraction method was optimized to simplify and accelerate the process, the RNA extraction step remains necessary, still incurring both time and cost. Lastly, the transition from laboratory settings to real field applications has not yet been achieved. In future, it could be considered to circumvent RNA extraction (e.g., direct RT-LAMP on crude lysates) in SMV testing to reduce the time and cost. In addition, future research could focus on integrating simpler sample handling methods and portable detection devices with the established RT-LAMP-LFD assay, which would enhance its utility for direct field testing.

In conclusion, this study developed a simple, rapid, portable, and robust assay for SMV-SC7 detection by combining MB-based nucleic acid extraction with the RT-LAMP-LFD approach. The method offers good specificity and high sensitivity, facilitating easy and intuitive detection of SMV-SC7 without the need of expensive instrumentation. RT-LAMP-LFD reduced the time by ~46% compared to RT-qPCR. This provides essential technical support for SMV control and early diagnosis of soybeans susceptible to SMV-SC7 infection. Additionally, the assay holds significant potential to serve as an ideal diagnostic tool for rapid and precise detection of SMV-SC7 infection in resource-limited settings.

## Data Availability

The datasets presented in this study can be found in online repositories. The names of the repository/repositories and accession number(s) can be found in the article/Supplementary material.

## References

[B1] Andrade T. P. D. Lightner D. V. (2009). Development of a method for the detection of infectious myonecrosis virus by reverse-transcription loop-mediated isothermal amplification and nucleic acid lateral flow hybrid assay. J. Fish Dis. 32, 911–924. 10.1111/j.1365-2761.2009.01072.x 19531063

[B2] Behrmann O. Bachmann I. Spiegel M. Schramm M. Wahed A. Dobler G. . (2020). Rapid detection of SARS-CoV-2 by low volume real-time single tube reverse transcription recombinase polymerase amplification using an Exo probe with an Internally linked quencher (Exo-IQ). Clin. Chem. 66, 10478–11054. 10.1093/clinchem/hvaa116 32384153 PMC7239256

[B3] Bhat A. I. Aman R. Mahfouz M. (2022). Onsite detection of plant viruses using isothermal amplification assays. Plant Biotechnol. J. 20, 1859–1873. 10.1111/pbi.13871 35689490 PMC9491455

[B4] Bhat A. I. Siljo A. Deeshma K. P. (2013). Rapid detection of Piper yellow mottle virus and *cucumber mosaic virus* infecting black pepper (*Piper nigrum*) by loop-mediated isothermal amplification (LAMP). J. Virol. Methods 193, 190–196. 10.1016/j.jviromet.2013.06.012 23791964

[B5] Branch D. W. Vreeland E. C. McClain J. L. Murton J. K. James C. D. Achyuthan K. E. (2017). Rapid nucleic acid extraction and purification using a miniature ultrasonic echnique. Micromachines 8:228. 10.3390/mi8070228 30400419 PMC6190382

[B6] Chen X. Zhou Q. X. Yuan W. Shi Y. F. Dong S. L. Luo X. H. (2023). Visual and rapid identification of *Chlamydia trachomatis* and *Neisseria gonorrhoeae* using multiplex loop-mediated isothermal amplification and a gold nanoparticle-based lateral flow biosensor. Front. Cell. Infect. Microbiol. 13:1067554. 10.3389/fcimb.2023.1067554 36926514 PMC10011439

[B7] Debreczeni D. E. Ruiz-Ruiz S. Aramburu J. López C. Belliure B. Galipienso L. . (2011). Detection, discrimination and absolute quantitation of *tomato spotted wilt virus* isolates using real time RT-PCR with TaqMan^®^MGB probes. J. Virol. Methods 176, 32–37. 10.1016/j.jviromet.2011.05.027 21635923

[B8] Dong Y. J. Zhao Y. J. Li S. W. Wan Z. Z. Lu R. F. Yang X. G. . (2022). Multiplex, real-time, point-of-care RT-LAMP for SARS-CoV-2 detection using the HFman probe. ACS Sens. 7, 730–739. 10.1021/acssensors.1c02079 35192340 PMC8887655

[B9] Faggi E. Pini G. Campisi E. (2005). Use of magnetic beads to extract fungal DNA. Mycoses 48, 3–7. 10.1111/j.1439-0507.2004.01030.x 15679657

[B10] Faye L. Grünwald-Gruber C. Vezina L. P. Gomord V. Morel B. (2024). A fast and easy one-step purification strategy for plant-made antibodies using Protein A magnetic beads. Front. Plant Sci. 14:1276148. 10.3389/fpls.2023.1276148 38235198 PMC10791999

[B11] Fei Z. J. Cheng C. Wei R. B. Tan G. L. Xiao P. F. (2022). Reversible superhydrophobicity unyielding magnetic beads of flipping-triggered (SYMBOL) regulate the binding and unbinding of nucleic acids for ultra-sensitive detection. Chem. Eng. J. 431:133953. 10.1016/j.cej.2021.133953

[B12] Fox A. Mumford R. A. (2017). Plant viruses and viroids in the United Kingdom: an analysis of first detections and novel discoveries from 1980 to 2014. Virus Res. 241, 10–18. 10.1016/j.virusres.2017.06.029 28690070

[B13] Ge X. Asiri A. M. Du D. Wen W. Wang S. Lin Y. (2014). Nanomaterial-enhanced paper-based biosensors. TrAC Trend. Anal. Chem. 58, 31–39. 10.1016/j.trac.2014.03.008

[B14] Ghosh D. K. Kokane S. B. Gowda S. (2020). Development of a reverse transcription recombinase polymerase based isothermal amplification coupled with lateral flow immunochromatographic assay (CTV-RT-RPA-LFICA) for rapid detection of *Citrus tristeza virus*. Sci. Rep. 10:20593. 10.1038/s41598-020-77692-w 33244066 PMC7693335

[B15] Ghosh D. K. Kokane S. B. Kokane A. D. Warghane A. J. Motghare M. R. Bhose S. . (2018). Development of a recombinase polymerase based isothermal amplification combined with lateral flow assay (HLB-RPA-LFA) for rapid detection of “*Candidatus Liberibacter asiaticus*.” *PLoS ONE* 13:0208530. 10.1371/journal.pone.0208530 30540789 PMC6291142

[B16] Hajimorad M. R. Domier L. L. Tolin S. A. Whitham S. A. Saghai Maroof M. A. (2018). *Soybean mosaic virus*: a successful potyvirus with a wide distribution but restricted natural host range. Mol. Plant Pathol. 19, 1563–1579. 10.1111/mpp.12644 29134790 PMC6638002

[B17] He Y. G. Xie T. Tong Y. G. (2021). Rapid and highly sensitive one-tube colorimetric RT-LAMP assay for visual detection of SARS-CoV-2 RNA. Biosens. Bioelectron. 187:113330. 10.1016/j.bios.2021.113330 34022500 PMC8117486

[B18] Iwamoto T. Sonobe T. Hayashi K. (2003). Loop-mediated isothermal amplification for direct detection of *Mycobacterium tuberculosis* complex, *M. avium*, and *M. intracellulare* in sputum samples. J. Clin. Microbiol. 41, 2616–2622. 10.1128/JCM.41.6.2616-2622.2003 12791888 PMC156570

[B19] Klein S. Müller T. G. Khalid D. Sonntag-Buck V. Heuser A. M. Glass B. . (2020). SARS-CoV-2 RNA extraction using magnetic beads for rapid large-scale testing by RT-qPCR and RT-LAMP. Viruses 12:863. 10.3390/v12080863 32784757 PMC7472728

[B20] Kokane A. Kokane S. Warghane A. Gubyad M. G. Sharma A. K. Reddy K. M. . (2020). A rapid and sensitive reverse transcription-loop-mediated isothermal amplification (RT-LAMP) assay for the detection of *Indian Citrus Ringspot Virus*. Plant Dis. 105, 1346–1355. 10.1094/PDIS-06-20-1349-RE 32990524

[B21] Larremore D. B. Wilder B. Lester E. Shehata S. Burke J. M. Hay J. A. . (2021). Test sensitivity is secondary to frequency and turnaround time for COVID-19 screening. Sci. Adv. 7:eabd5393. 10.1126/sciadv.abd5393 33219112 PMC7775777

[B22] Lee J. E. Kim S. A. Park H. J. Mun H. Ha K. S. Shim W. B. (2022). Colorimetric detection of norovirus by helicase-dependent amplification method based on specific primers integrated with HRPzyme. Anal. Bioanal. Chem. 414, 6723–6733. 10.1007/s00216-022-04247-5 35931785

[B23] Lee S. Khoo V. S. L. Medriano C. A. D. Lee T. Park S. Y. Bae S. (2019). Rapid and *in-situ* detection of fecal indicator bacteria in water using simple DNA extraction and portable loop-mediated isothermal amplification (LAMP) PCR methods. Water Res. 160, 371–379. 10.1016/j.watres.2019.05.049 31163314

[B24] Li B. Mou X. B. Chen Z. Chen H. Deng Y. Li S. . (2017). The development of a rapid high-quality universal nucleic acid extraction kit based on magnetic separation. Sci. China Chem. 60, 1602–1608. 10.1007/s11426-017-9061-1

[B25] Li C. Guo S. X. Sun M. Niu J. P. Yin C. C. Du W. J. . (2024). A colorimetric RT-LAMP assay for rapid detection of *soybean mosaic virus* SC15. ACS Omega 9, 29765–29775. 10.1021/acsomega.4c03372 39005798 PMC11238210

[B26] Liu J. Z. Fang Y. Pang H. X. (2016). The current status of the soybean-*soybean mosaic virus* (SMV) *pathosystem*. Front. Microbiol. 7:01906. 10.3389/fmicb.2016.01906 27965641 PMC5127794

[B27] Lizardi P. M. Ward D. C. Huang X. Zhu Z. Bray-Ward P. Thomas D. C. (1998). Mutation detection and single-molecule counting using isothermal rolling-circle amplification. Nat. Genet. 19, 225–232. 10.1038/898 9662393

[B28] Luan H. X. Liao W. L. Niu H. P. Cui X. Y. Chen X. Zhi H. J. (2019). Comprehensive analysis of *soybean mosaic virus* P3 protein interactors and hypersensitive response-like lesion-inducing protein function. Int. J. Mol. Sci. 20:3388. 10.3390/ijms20143388 31295900 PMC6678280

[B29] Luan H. X. Shine M. B. Cui X. Y. Chen X. Ma N. Kachroo P. . (2016). The potyviral P3 protein targets eukaryotic elongation factor 1A to promote the unfolded protein response and viral pathogenesis. Plant Physiol. 172, 221–234. 10.1104/pp.16.00505 27356973 PMC5074642

[B30] Luo G. C. Yi T. T. Wang Q. Guo B. Fang L. Zhang G. Y. . (2021). Stem-loop-primer assisted isothermal amplification enabling high-specific and ultrasensitive nucleic acid detection. Biosens. Bioelectron. 184:113239. 10.1016/j.bios.2021.113239 33857727

[B31] Mao K. Zhang H. Ran F. Cao H. R. Feng R. D. Du W. . (2024). Portable biosensor combining CRISPR/Cas12a and loop-mediated isothermal amplification for antibiotic resistance gene ermB in wastewater. J. Hazard. Mater. 462:132793. 10.1016/j.jhazmat.2023.132793 37856955

[B32] Nakiboneka R. Walbaum N. Musisi E. Nevels M. Nyirenda T. Nliwasa M. . (2024). Specific human gene expression in response to infection is an effective marker for diagnosis of latent and active tuberculosis. Sci. Rep. 14:26884. 10.1038/s41598-024-77164-5 39505948 PMC11541504

[B33] Nimitphak T. Kiatpathomchai W. Flegel T. W. (2008). Shrimp hepatopancreatic parvovirus detection by combining loop-mediated isothermal amplification with a lateral flow dipstick. J. Virol. Methods 154, 56–60. 10.1016/j.jviromet.2008.09.003 18835299

[B34] Notomi T. Okayama H. Masubuchi H. Yonekawa T. Watanabe K. Amino N. . (2000). Loop-mediated isothermal amplification of DNA. Nucleic Acids Res. 28:e63. 10.1093/nar/28.12.e63 10871386 PMC102748

[B35] Palani S. N. Elangovan S. Menon A. Kumariah M. Tennyson J. (2019). An efficient nucleic acids extraction protocol for *Elettaria cardamomum*. Biocatal. Agri. Biotechnol. 17, 207–212. 10.1016/j.bcab.2018.11.026

[B36] Paul R. Ostermann E. Wei Q. S. (2020). Advances in point-of-care nucleic acid extraction technologies for rapid diagnosis of human and plant diseases. Biosens. Bioelectron. 169:112592. 10.1016/j.bios.2020.112592 32942143 PMC7476893

[B37] Peng Q. D. Ning J. C. Xu Q. Y. Yang T. Wang Y. R. Zheng T. R. . (2021). Development and application of a reverse transcription loop-mediated isothermal amplification combined with lateral flow dipstick for rapid and visual detection of *Citrus leaf blotch virus* in kiwifruit. Crop Prot. 143:105555. 10.1016/j.cropro.2021.105555

[B38] Rahman A. M. A. Ransangan J. Subbiah V. K. (2022). Improvements to the rapid detection of the marine pathogenic bacterium, *Vibrio harveyi*, using loop-mediated isothermal amplification (LAMP) in combination with SYBR Green. Microorganisms 10:2346. 10.3390/microorganisms10122346 36557599 PMC9786892

[B39] Rubio L. Galipienso L. Ferriol I. (2020). Detection of plant viruses and disease management: relevance of genetic diversity and evolution. Front. Plant Sci. 11:01092. 10.3389/fpls.2020.01092 32765569 PMC7380168

[B40] Sheu S. C. Tsou P. C. Lien Y. Y. Lee M. S. (2018). Development of loop-mediated isothermal amplification (LAMP) assays for the rapid detection of allergic peanut in processed food. Food Chem. 257, 67–74. 10.1016/j.foodchem.2018.02.124 29622231

[B41] Sheveleva A. Ivanov P. Gasanova T. Osipov G. Chirkov S. (2018). Sequence analysis of *plum pox virus* strain C isolates from Russia revealed prevalence of the D96E mutation in the universal epitope and interstrain recombination events. Viruses 10:450. 10.3390/v10090450 30142962 PMC6164383

[B42] Simões A. E. S. Pereira D. M. Amaral J. D. Nunes A. F. Gomes S. E. Rodrigues P. M. . (2013). Efficient recovery of proteins from multiple source samples after trizol^®^ or trizol^®^LS RNA extraction and long-term storage. BMC Genomics 14:181. 10.1186/1471-2164-14-181 23496794 PMC3620933

[B43] Singh M. D. Singh H. Singh N. K. Singh N. K. Kashyap N. Sood N. K. . (2019). Development of loop-mediated isothermal amplification (LAMP) assay for detection of *Hepatozoon canis* infection in dogs. Ticks Tick-borne Dis. 10, 371–376. 10.1016/j.ttbdis.2018.11.016 30503892

[B44] Smuts H. Kew M. Khan A. Korsman S. (2014). Novel hybrid parvovirus-like virus, NIH-CQV/PHV, contaminants in silica column-based nucleic acid extraction kits. J. Virol. 88, 1398–1398. 10.1128/JVI.03206-13 24335290 PMC3911631

[B45] Song S. Wang J. Zhou J. Y. Cheng X. F. Hu Y.X. Wang J. H. . (2024). Single-Cell RNA-sequencing of soybean reveals transcriptional changes and antiviral functions of GmGSTU23 and GmGSTU24 in response to soybean mosaic virus. Plant Cell Environ. 10.1111/pce.15164 39301882

[B46] Thongkao K. Longyant S. Silprasit K. Sithigorngul P. Chaivisuthangkura P. (2015). Rapid and sensitive detection of *Vibrio harveyi* by loop-mediated isothermal amplification combined with lateral flow dipstick targeted to vhhP2 gene. Aquac. Res. 46, 1122–1131. 10.1111/are.1226621513793

[B47] Urcuqui-Inchima S. Haenni A.-L. Bernardi F. (2001). Potyvirus proteins: a wealth of functions. Virus Res. 74, 157–175. 10.1016/S0168-1702(01)00220-9 11226583

[B48] Usovsky M. Chen P. Y. Li D. X. Wang A. M. Shi A. N. Zheng C. M. . (2022). Decades of genetic research on *soybean mosaic virus* resistance in soybean. Viruses 14:1122. 10.3390/v14061122 35746594 PMC9230979

[B49] Valiant W. G. Borman J. Cai K. Vallone P. M. (2024). Efficient extraction of adventitious virus nucleic acid using commercially available methods. Biologicals 85:101741. 10.1016/j.biologicals.2023.101741 38157678

[B50] Vincent M. Xu Y. Kong H. (2004). Helicase-dependent isothermal DNA amplification. EMBO Rep. 5, 795–800. 10.1038/sj.embor.7400200 15247927 PMC1249482

[B51] Wang J. H. Ali Z. Wang N. Y. Liang W. B. Liu H. N. Li F. . (2015). Simultaneous extraction of DNA and RNA from *Escherichia coli* BL 21 based on silica-coated magnetic nanoparticles. Sci. China Chem. 58, 1774–1778. 10.1007/s11426-015-5483-x29634167

[B52] Wang N. Zhang J. Xiao B. Li H. Chen J. C. Sun X. Y. . (2023). Integration of in-cassette lysis, purification, and lateral flow strips-based sensor for rapid and on-site detection of yak milk adulteration. Sens. Actuators B Chem. 394:134309. 10.1016/j.snb.2023.134309

[B53] Wang Y. Dai J. F. Liu Y. S. Yang J. F. Hou Q. Ou Y. W. . (2021). Development of a potential penside colorimetric LAMP assay using neutral red for detection of *African swine fever virus*. Front. Microbiol. 12:609821. 10.3389/fmicb.2021.609821 33967972 PMC8102904

[B54] Wang Y. Zhao X. Zhou Y. H. Lu J. R. Yu H. L. Li S. J. (2022). Establishment and application of loop-mediated isothermal amplification coupled with nanoparticle-based lateral flow biosensor (LAMP-LFB) for visual and rapid diagnosis of *Candida albicans* in clinical samples. Front. Bioeng. Biotech. 10:1025083. 10.3389/fbioe.2022.1025083 36420441 PMC9676452

[B55] Wang Z. Y. Ge W. X. Bi W. T. Chen D. D. Y. (2024). Strategies for using magnetic beads in enhanced deep eutectic solvent-mechanochemical extraction of natural products from orange peels. Food Chem. 447:139004. 10.1016/j.foodchem.2024.139004 38492304

[B56] Wharam S. D. Marsh P. Lloyd J. S. Ray T. D. Mock G. A. Assenberg R. . (2001). Specific detection of DNA and RNA targets using a novel isothermal nucleic acid amplification assay based on the formation of a three-way junction structure. Nucleic Acids Res. 29:e54. 10.1093/nar/29.11.e54 11376166 PMC55724

[B57] Wielinga P. R. de Heer L. de Groot A. Hamidjaja R. A. Bruggeman G. Jordan K. . (2011). Evaluation of DNA extraction methods for *Bacillus anthracis* spores spiked to food and feed matrices at biosafety level 3 conditions. Int. J. Food Microbiol. 150, 122–127. 10.1016/j.ijfoodmicro.2011.07.023 21864928

[B58] Wu W. H. Wang G. H. Wang H. Gbokie T. He C. Huang X. . (2024). Development of a loop-mediated isothermal amplification assay for rapid and sensitive detection of *Hemileia vastatrix* in coffee plantations. Trop. Plant Pathol. 49, 515–524. 10.1007/s40858-023-00627-z

[B59] Wu W. J. Yin C. C. Yue A. Q. Niu J. P. Du W. J. Liu D. B. . (2022). Rapid and visual detection of *soybean mosaic virus* SC7 with a loopmediated isothermal amplification strategy. Sens. Actuators B. 373:132733. 10.1016/j.snb.2022.132733

[B60] Xu K. J. Wang Y. Z. Zhang H. M. Yang Q. Wei X. X. Xu P. L. . (2017). Solid-phase extraction of DNA by using a composite prepared from multiwalled carbon nanotubes, chitosan, Fe3O4 and a poly (ethylene glycol)-based deep eutectic solvent. Microchim. Acta 184, 4133–4140. 10.1007/s00604-017-2444-4

[B61] Yang C. Zhen Y. Hou J. L. Mi T. Z. (2024). Development of a rapid detection method to *Prorocentrum lima* by loop-mediated isothermal amplification with hydroxy naphthol blue. Mar. Biotechnol. 26, 475–487. 10.1007/s10126-024-10310-2 38602600

[B62] Yi H. W. Wang X. M. Tan X. Ding C. Z. Zhang C. L. Wu J. H. . (2024). Simultaneous detection of human norovirus GI, GII and SARS-CoV-2 by a quantitative one-step triplex RT-qPCR. Front. Microbiol. 14:1269275. 10.3389/fmicb.2023.1269275 38260899 PMC10800780

[B63] Zeng L. Y. Su Y. Stejskal V. Opit G. Aulicky R. Li Z. H. (2021). Primers and visualization of LAMP: a rapid molecular identification method for *Liposcelis entomophila* (Enderlein) (Psocodea: Liposcelididae). *J. Stored Prod. Res*. 93:101855. 10.1016/j.jspr.2021.101855

[B64] Zhang F. Wu J. Wang R. Wang L. Ying Y. B. (2014). Portable pH-inspired electrochemical detection of DNA amplification. Chem. Commun. 50, 8416–8419. 10.1039/c4cc03011g 24947973

[B65] Zhang K. Shen Y. C. Wang T. Wang Y. Xue S. Luan H. X. . (2022). GmGSTU13 is related to the development of mosaic symptoms in soybean plants infected with *soybean mosaic virus*. Phytopathology 112, 452–459. 10.1094/PHYTO-11-20-0498-R 34077233

[B66] Zhang L. Deraney R. N. Tripathi A. (2015). Adsorption and isolation of nucleic acids on cellulose magnetic beads using a three-dimensional printed microfluidic chip. Biomicrofluidics 9:064118. 10.1063/1.4938559 26734116 PMC4693444

[B67] Zhang Z. M. Zhao S. H. Jiang L. J. Wu J. J. Zhao W. H. Guo X. N. . (2022). A sample-to-answer DNA detection microfluidic system integrating sample pretreatment and smartphone-readable gradient plasmonic photothermal continuous-flow PCR. Analyst 147, 4876–4887. 10.1039/D2AN00908K 36155591

