# Supplementary material for: Swift and portable detection of soybean mosaic virus SC7 through RNA extraction and loop-mediated isothermal amplification using lateral flow device
Source: Front Microbiol. 2025 Jan 3;15:1478218. doi: 10.3389/fmicb.2024.1478218 (PMC11739293; doi:10.3389/fmicb.2024.1478218)
Supplement: Supplementary file 1 [file Data_Sheet_1.PDF]

Supplementary Material

Supplementary Table 1. The sequences of LAMP primers

Primer name	Primer sequence (5'-3')
SC7- FIP	CCTCTTGGTGAGCTGTTCCAAGATAAGTAAGGAACATAGTGTGG
SC7- BIP	CTGCAAATGACGTGCTACTTGAGGACAGTCCTCTAATATGCT
SC7- F3	AAAAGGGGTGGAGTTGTG
SC7- B3	CTTGTATGACGGTGGTACT
SC7- LB	GCAGCTTGAAATGATTTCAGAGAC
SC7- BIP-Biotin	Biotin-CTGCAAATGACGTGCTACTTGAGGACAGTCCTCTAATATGCT
SC7- LB-FAM	FAM-GCAGCTTGAAATGATTTCAGAGAC

Supplementary Table 2. The detection of 194 field samples was performed using RT-qPCR, colorimetric LAMP, and RT-LAMP-LFD.

ID	variety	RT-qPCR	Colorimetric RT-LAMP	RT-LAMP-LFD
1	SZ1283	-	-	-
2	SZ1298	+	+	+
3	SZ1327	+	+	+
4	SZ1330	-	-	-
5	SZ1331	-	-	-
6	SZ1455	-	-	-

7	SZ1456	-	-	-
8	SD17	-	-	-
9	S936	-	-	-
10	SZ1344	-	-	-
11	SZ1347	-	+	-
12	SZ1353	-	-	-
13	SZ1362	+	+	+
14	QD2014	-	-	-
15	JD37	-	-	-
16	JD47	-	-	-
17	JD118	-	-	-
18	JD116	+	-	+
19	JD53	-	+	-
20	JD140	-	-	-
21	JD134	-	-	-
22	SZ220-1	+	+	+
23	SZ350	-	-	-
24	SZ292	-	-	-
25	SZ355	-	-	-
26	SZ747	-	-	-
27	SZ750	-	-	-
28	SZ1083	+	+	+
29	SZ1112	-	-	-
30	SZ1144	+	+	+
31	SZ1297	+	+	+

32	SZ1348	-	-	-
33	SZ288	-	-	-
34	SZ351	-	-	-
35	SZ358	-	-	-
36	SZ693	-	-	-
37	SZ850	-	+	-
38	SZ919	-	-	-
39	SZ1113	-	-	-
40	SZ1135	-	-	-
41	SZ1139	-	-	-
42	SZ1308	-	-	-
43	SZ1363	-	-	-
44	SZ1410	+	+	+
45	SZ829	+	+	+
46	SZ874	+	-	+
47	SZ865	-	-	-
48	SZ883	-	-	-
49	SZ885	-	-	-
50	SZ907	+	+	+
51	SZ916	+	+	+
52	SZ962	-	-	-
53	SZ1023	-	-	-
54	SZ1070	+	+	+
55	SZ1081	+	+	+
56	SZ1084	+	-	-

57	SZ1111	+	+	+
58	SZ1114	+	-	+
59	SZ1118	+	+	+
60	SZ1128	-	-	-
61	SZ1303	+	+	+
62	SZ1317	-	-	-
63	SD25	-	-	-
64	SZ1358	+	+	+
65	JD131	-	-	-
66	JD142	-	-	-
67	JD143	-	-	-
68	SZ1424	-	-	-
69	SZ847	-	-	-
70	SZ854	-	-	-
71	SZ856	-	-	-
72	SZ882	-	-	-
73	SZ888	-	-	-
74	SZ913	-	-	-
75	SZ922	+	+	+
76	SZ923	-	-	-
77	SZ967	-	-	-
78	SZ954	-	-	-
79	SZ981	-	-	-
80	SZ1022	-	-	-
81	SZ1040	-	-	-

82	SZ1041	-	-	-
83	SZ1067	-	-	-
84	SZ1069	-	-	-
85	SZ1136	-	-	-
86	SZ1152	-	-	-
87	SZ1175	-	-	-
88	SZ1251	+	-	+
89	SZ1355	-	-	-
90	SZ1357	-	-	-
91	SZ1408	+	+	+
92	SZ1413	+	-	+
93	SZ319	-	-	-
94	SZ353	+	-	+
95	SZ699	-	-	-
96	SZ864	-	-	-
97	SZ928	-	-	-
98	SZ933	-	-	-
99	SZ954	-	-	-
100	SZ983	-	-	-
101	SZ993	-	-	-
102	SZ1021	-	-	-
103	SZ1050	-	-	-
104	SZ1147	-	-	-
105	SZ1168	-	-	-
106	SZ1180	+	-	+

107	SZ1183	+	+	+
108	SZ1228	-	-	-
109	SZ1346	+	-	+
110	SZ1359	+	-	+
111	SZ1361	-	-	-
112	SZ1398	+	-	+
113	SZ1407	+	-	-
114	SZ1419	-	-	-
115	SZ1230	-	-	-
116	SZ916	+	+	+
117	SZ1053	-	-	-
118	SZ1159	+	-	+
119	SZ1160	+	+	+
120	SZ1164	-	-	-
121	SZ1166	-	-	-
122	SZ1172	+	-	+
123	SZ1185	-	-	-
124	SZ1186	-	-	-
125	SZ1197	+	+	+
126	SZ1199	-	-	-
127	SZ1202	-	-	-
128	SZ1229	-	-	-
129	SZ1239	-	-	-
130	SZ1244	+	-	-
131	SZ1293	-	-	-

132	SZ1294	-	-	-
133	SZ1295	+	+	+
134	SZ1296	-	-	-
135	SZ1299-1	-	-	-
136	ND5240	+	-	+
137	SZ1404	-	-	-
138	SZ1409	-	-	-
139	SZ1418	-	-	-
140	HB5	+	+	+
141	SZ17	-	-	-
142	SZ356	-	-	-
143	SZ357	-	-	-
144	SZ359	-	-	-
145	SZ855	-	-	-
146	SZ908	+	+	+
147	SZ924	-	-	-
148	SZ1123	-	-	-
149	SZ1130	-	-	-
150	SZ1133	+	+	+
151	SZ1225	+	-	+
152	SZ1300	+	+	+
153	SZ1310	+	-	+
154	SZ1322	+	+	+
155	SZ1326	-	-	-
156	SZ1328	+	+	+

157	SZ1421	+	+	+
158	石 885	-	-	-
159	SZ1356	-	-	-
160	SZ1360	-	-	-
161	SZ1402	-	-	-
162	SZ1417	-	-	-
163	HD13	+	+	+
164	JD114	+	-	+
165	SZ1042	-	-	-
166	SZ1072	+	-	+
167	SZ1171	-	-	-
168	SZ1284	-	-	-
169	SD12	+	+	+
170	JD19	+	-	+
171	JD136	-	-	-
172	JD141	-	-	-
173	SZ60	-	-	-
174	SZ844	+	+	+
175	SZ834	-	-	-
176	SZ910	-	-	-
177	SZ915	-	-	-
178	SZ929	-	-	-
179	SZ931	-	-	-
180	SZ989	+	+	+
181	SZ1073	+	-	+

182	SZ1080	-	-	-
183	SZ1132	-	-	-
184	SZ1176	+	+	+
185	SZ1195	-	-	-
186	SZ1277	-	-	-
187	SD14	+	-	+
188	SZ1429	+	+	+
189	SZ1351	-	-	-
190	JY53	-	-	-
191	KF1	-	-	-
192	SZ1411	-	-	-
193	JD117	+	-	+
194	JD133	+	+	+

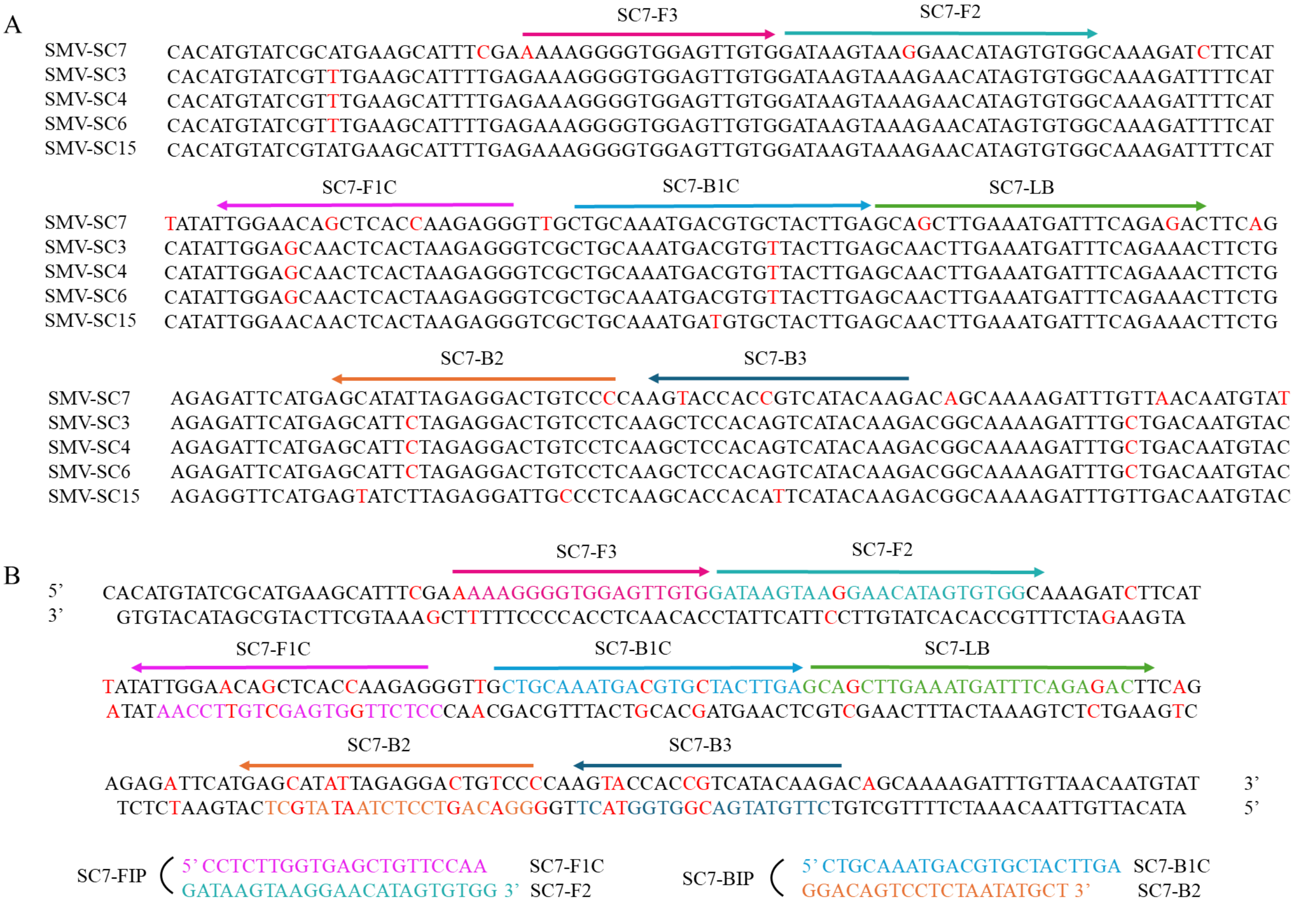

Supplementary Figure 1. Design of LAMP primer set for SMV-SC7 based on the conserved sequence of SMV-P3 gene. (A) Alignment of the P3 gene sequences across different SMV strains and design of the LAMP primer sites. (B) Primers for LAMP assays were designed.

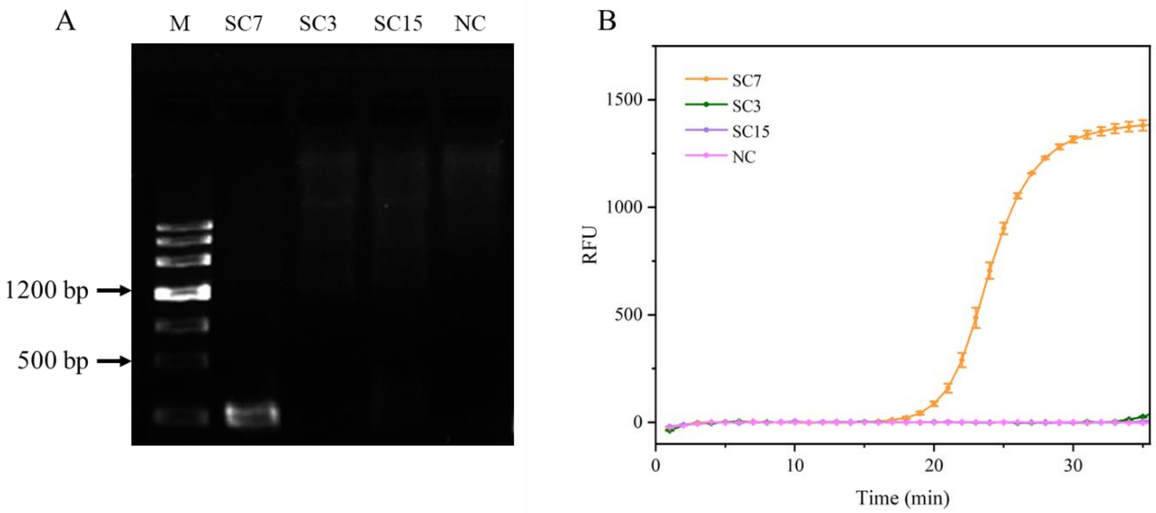

Supplementary Figure 2. Verification of the RT-qPCR assay specificity for SMV- SC 7. The cDNA of SMV-SC3, SMV-SC7, and SMV-SC15 was used to test the specificity of the RT-qPCR assay. NC: negative control.

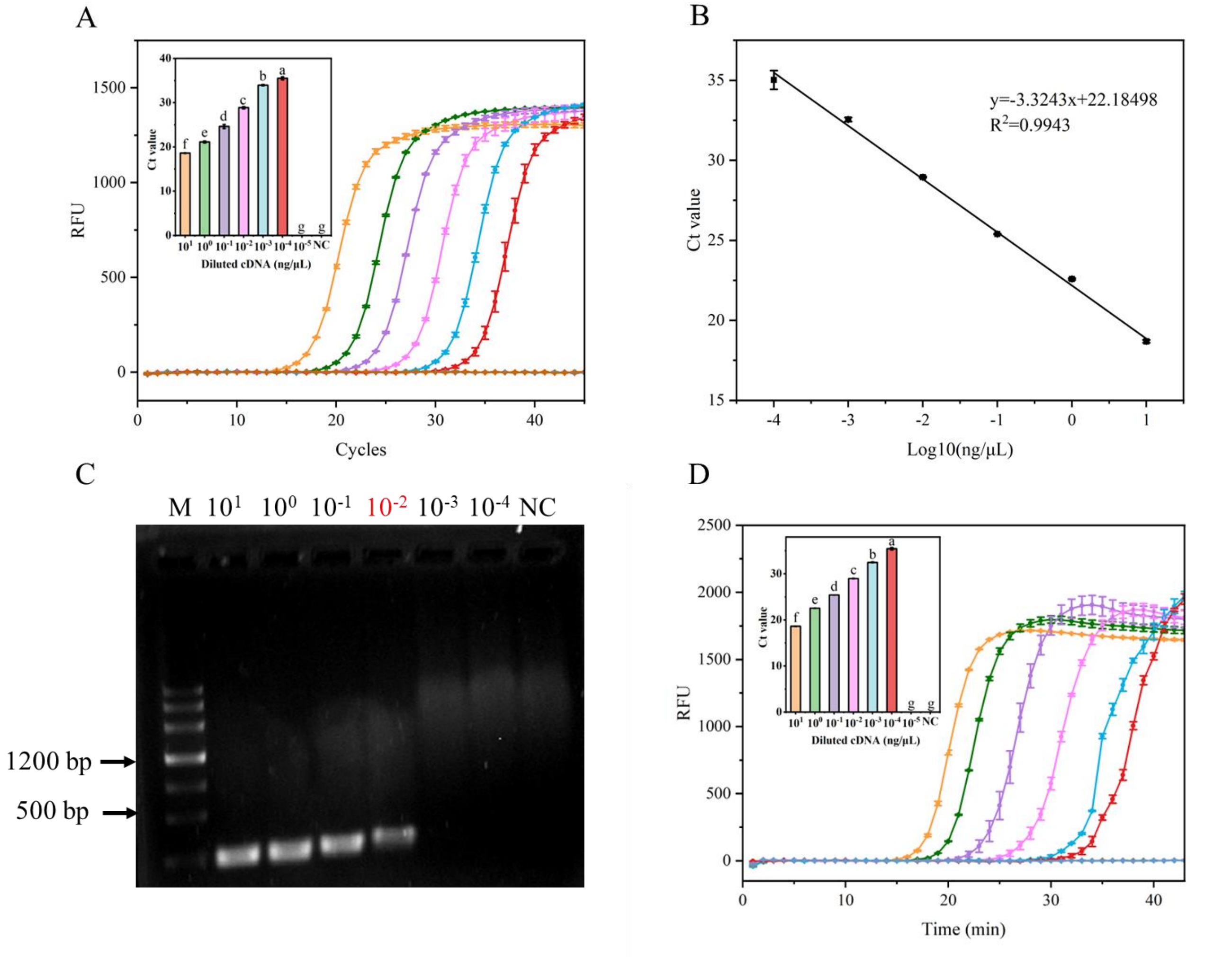

Supplementary Figure 3. (A) RT-qPCR detection of different concentrations of SMV-SC7 (10-fold dilution gradient) was performed. (B) A standard quantitative curve correlating various concentrations of SMV-SC7 with cycle threshold times (Ct) was developed. (C) Characterization of the *conventional PCR* product of SMV-SC7 by gel electrophoresis. (D) Real time fluorescence RT-LAMP detection of different concentrations of SMV-SC7 (10-fold dilution gradient) was performed. Error bars represent SD of mean, n=3. $P < 0.05$ was considered statistically significant. Concentrations of SMV-SC7 cDNA templates of 10^1, 10^0, 10^{-1}, 10^{-2}, 10^{-3}, 10^{-4}, 10^{-5}, and 10^{-6} ng/μL. M: DNA Marker III; NC: negative control.

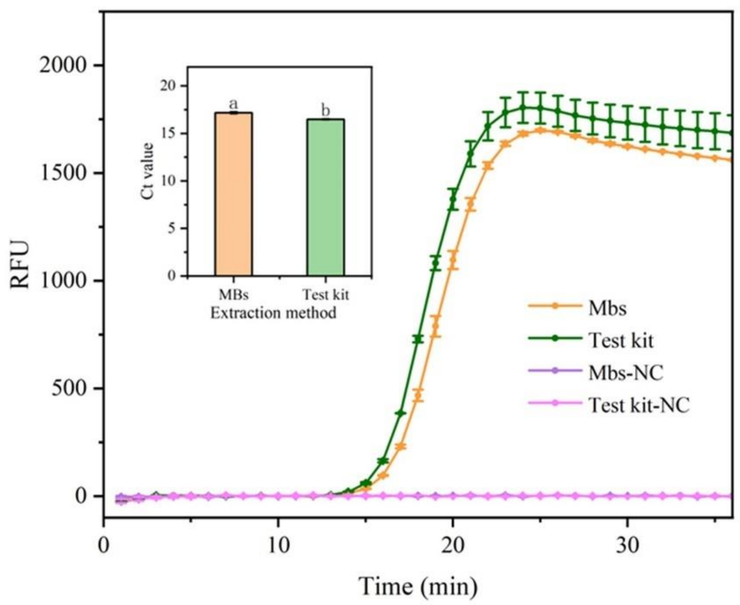

Supplementary Figure 4. Evaluates the performance of RNA extraction methods used MBs and commercial RNA extraction kit in LAMP fluorescence quantification.

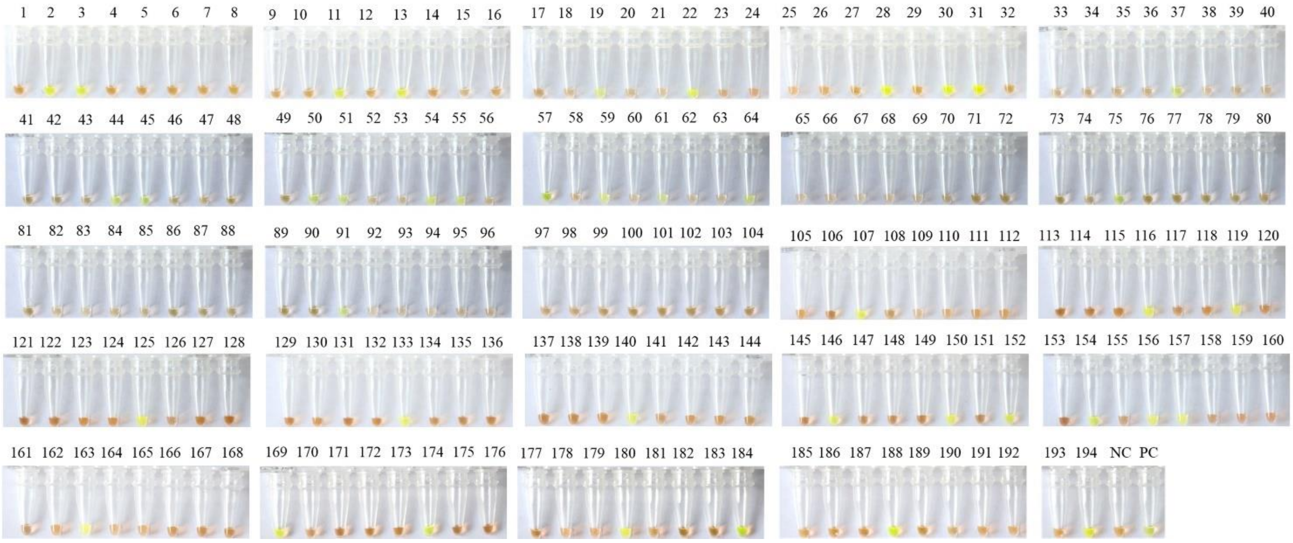

Supplementary Figure 5. Visual detection of RT-LAMP using SYBR Green I was performed.

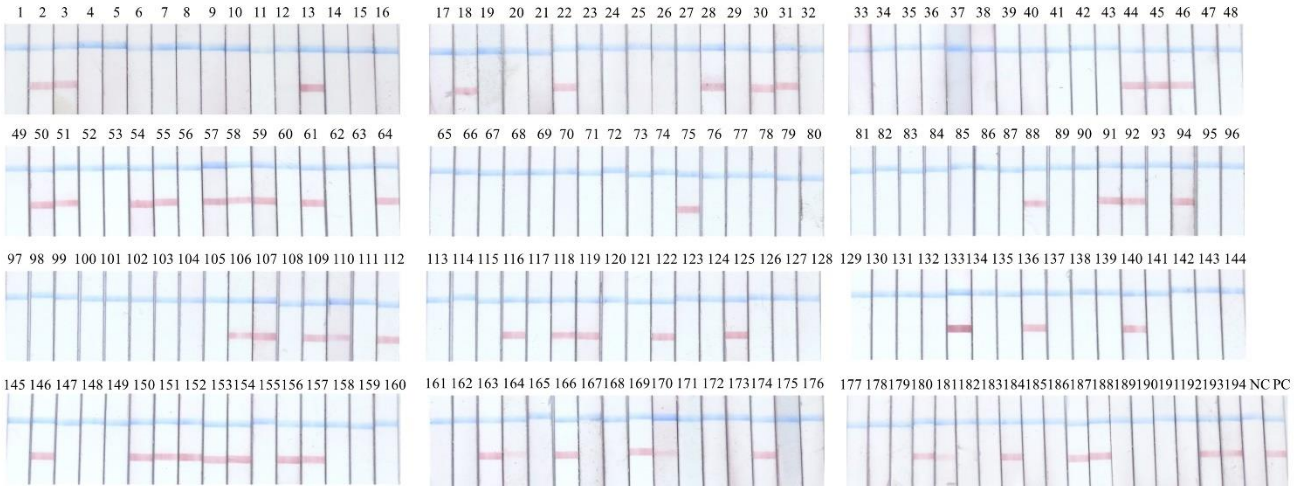

Supplementary Figure 6. Visual detection was conducted using the RT-LAMP-LFD method.
